# Discovery of Novel 2,4-Dianilinopyrimidine Derivatives Containing 4-(Morpholinomethyl)phenyl and *N*-Substituted Benzamides as Potential FAK Inhibitors and Anticancer Agents

**DOI:** 10.3390/molecules26144187

**Published:** 2021-07-09

**Authors:** Chun Han, Kemin Shen, Shijun Wang, Zhijun Wang, Feng Su, Xi Wu, Xiaoqin Hu, Mengyao Li, Jing Han, Lintao Wu

**Affiliations:** 1 Department of Chemistry, Changzhi University, Changzhi 046011, China; coldspringfibre@126.com (C.H.); 444795415@nwafu.edu.cn (S.W.); czxywzj@czc.edu.cn (Z.W.); sufeng@czc.edu.cn (F.S.); wuxi040252768@163.com (X.W.); xiaoqin_hu2021@126.com (X.H.); lmysummer@126.com (M.L.); 2 Department of Public Health and Preventive Medicine, Changzhi Medical College, Changzhi 046011, China; shenkm@czmc.edu.cn; 3 School of Chemistry and Materials Science, Jiangsu Normal University, Xuzhou 221116, China

**Keywords:** FAK inhibitor, 2,4-dianilinopyrimidine, 4-(morpholinomethyl)phenyl, *N*-substituted benzamides, anticancer, molecular docking

## Abstract

Focal adhesion kinase (FAK) is responsible for the development and progression of various malignancies. With the aim to explore novel FAK inhibitors as anticancer agents, a series of 2,4-dianilinopyrimidine derivatives **8a**–**8i** and **9a**–**9g** containing 4-(morpholinomethyl)phenyl and *N*-substituted benzamides have been designed and synthesized. Among them, compound **8a** displayed potent anti-FAK activity (IC_50_ = 0.047 ± 0.006 μM) and selective antiproliferative effects against H1975 (IC_50_ = 0.044 ± 0.011 μM) and A431 cells (IC_50_ = 0.119 ± 0.036 μM). Furthermore, compound **8a** also induced apoptosis in a dose-dependent manner, arresting the cells in S/G2 phase and inhibiting the migration of H1975 cells, all of which were superior to those of TAE226. The docking analysis of compound **8a** was performed to elucidate its possible binding modes with FAK. These results established **8a** as our lead compound to be further investigated as a potential FAK inhibitor and anticancer agent.

## 1. Introduction

Cancer is one of the most dreaded diseases impacting human health, and is one of the leading causes of death throughout the world today. In 2020, an estimated 19.3 million new cancer cases and almost 10.0 million cancer deaths were reported worldwide [1]. For cancer patients, traditional cytotoxic chemotherapy has been the most common treatment method, but it suffers from a lack of selectivity for tumor cells and frequently causes drug resistance during the course of treatment [2,3]. However, in recent years, there has been a significant emphasis on the development of targeted cancer therapies that are designed to enhance efficacy and selectivity for the treatment of various cancers by targeting specific cancer biomarkers [3,4,5].

Focal adhesion kinase (FAK), also known as protein tyrosine kinase 2 (PTK2), is a 125 kDa non-receptor tyrosine kinase protein that is involved in a variety of cellular processes, such as cross-linked signaling events mediated by integrins, stimulating the dissociation of G protein-coupled receptor subunits, and regulating the production of growth factors and neurotransmitters, all of which affect cell adhesion, motility, migration, apoptosis, and cell–matrix interactions [6]. FAK plays a crucial role in the development and progression of cancer, and its upregulation or overexpression has been frequently observed in a variety of malignancies, including colorectal, breast [7], thyroid [8], prostate [9], brain [10], ovarian [11], liver [12], stomach [13], and lung cancers [14]. In addition, FAK has been implicated in the development of chemoresistance. For example, ovarian cancer cells have been shown to confer resistance to platinum-based chemotherapies by increasing FAK activity needed to sustain cellular vitality and growth [15]. In addition, targeting FAK in endothelial cells was found to sensitize tumor cells to DNA-damaging therapies, which implicated FAK in the regulation of tumor chemosensitivity [16]. Other recent studies have revealed that FAK mediated immune responses to certain cancers. Inhibition of FAK also induced T cell-mediated tumor regression in mice, corroborating the potential to modulate FAK activity as a means to control antitumor immunity [17,18]. However, low-dose FAK inhibition was reported to increase tumor growth and angiogenesis, which was contrary to higher FAK inhibition, indicating that FAK can have a nonlinear dose-dependent function in regulating tumor growth and angiogenesis [19]. Nevertheless, FAK represents a promising biological target for the development of effective new anticancer therapies.

**Figure 1:**
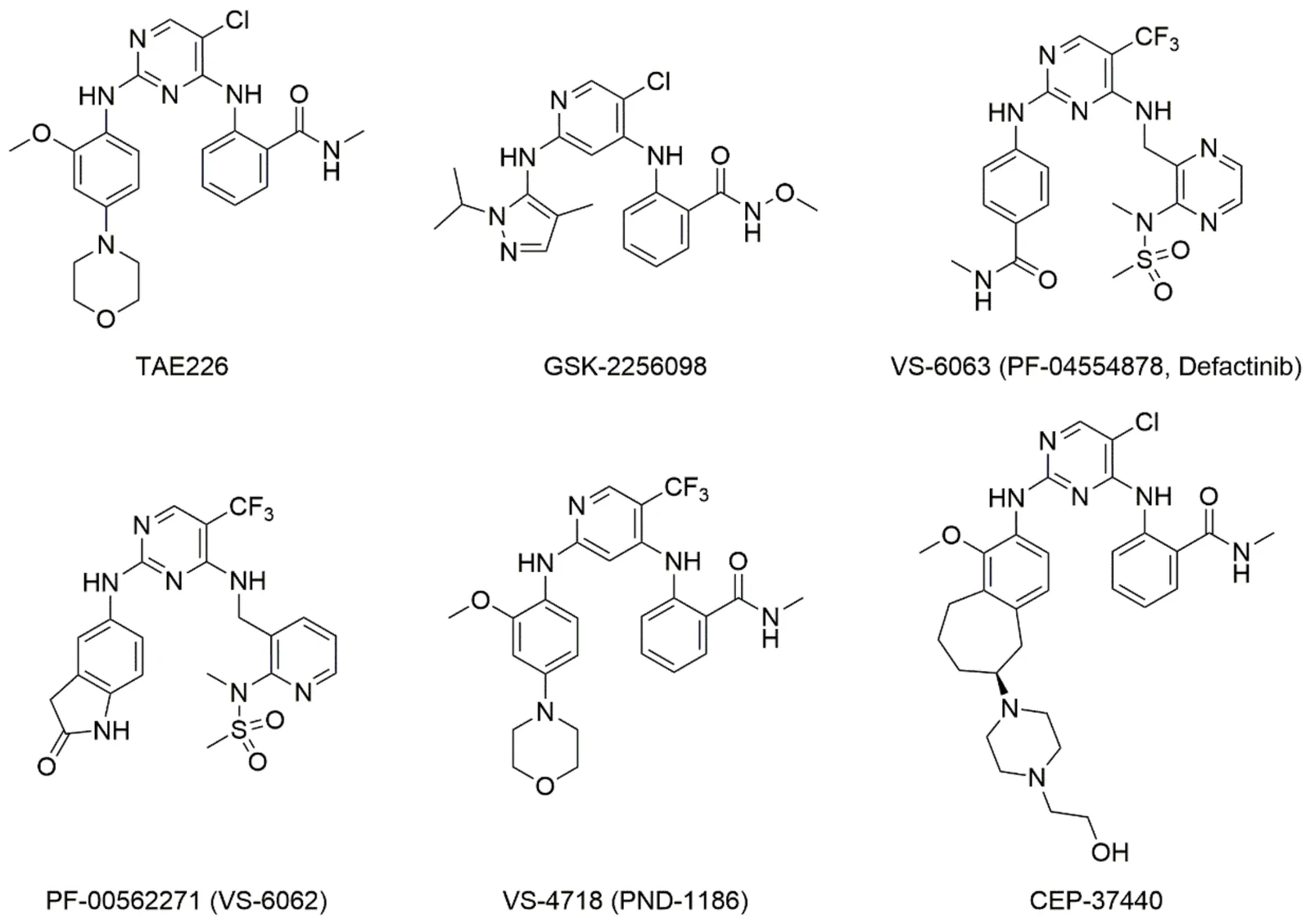
Structures of ATP-competitive FAK inhibitors in preclinical and clinical trials.

The FAK protein comprises a multidomain structure featuring an N-terminal FERM domain, a kinase domain, and a C-terminal domain. Targeting the ATP binding pocket in the kinase domain using small molecules has been the most common approach toward FAK inhibition. Several ATP-competitive FAK inhibitors (Figure 1) have been successfully developed, with some of them, including TAE226, GSK-2256098, VS-6063 (PF-04554878, Defactinib), PF-00562271 (VS-6062), VS-4718 (PND-1186), CEP-37440, and BI-853520 (IN-10018), progressing through preclinical development and even clinical trials [20]. These compounds displayed potent inhibition against FAK and demonstrated antitumor effects both in vitro and in vivo in many different malignancy models. To date, GSK-2256098 [21], PF-00562271 [22], CEP-37440 [23], and BI-853520 [24] have successfully completed their Phase I clinical trials, and VS-6063 [25] recently completed its Phase II clinical trial. However, the clinical trial of VS-4718 [26] was terminated or withdrawn, and TAE226 [27] was abandoned due to the discovery of several off-target effects, though TAE226 is extensively employed as a positive control to compare to other newly developed FAK inhibitors. Due to the lack of marketed FAK inhibitors, there is an urgent need to develop novel FAK inhibitors with effective antitumor activity. The majority of novel FAK inhibitors currently in development comprise a 2,4-diaminopyrimidine core, which was found to be an important structural moiety for promoting the binding of the inhibitors in the hinge region of the ATP binding site of FAK, as the compounds are stabilized through hydrogen bonds between active site residues and the pyrimidine nitrogen and aniline NH of the 2,4-diaminopyrimidine core [28,29]. Therefore, the 2,4-diaminopyrimidine is an attractive scaffold from which small-molecule inhibitors against FAK can be designed to improve the inhibition activity against FAK and other physicochemical properties compared to current inhibitors in development.

**Figure 2:**
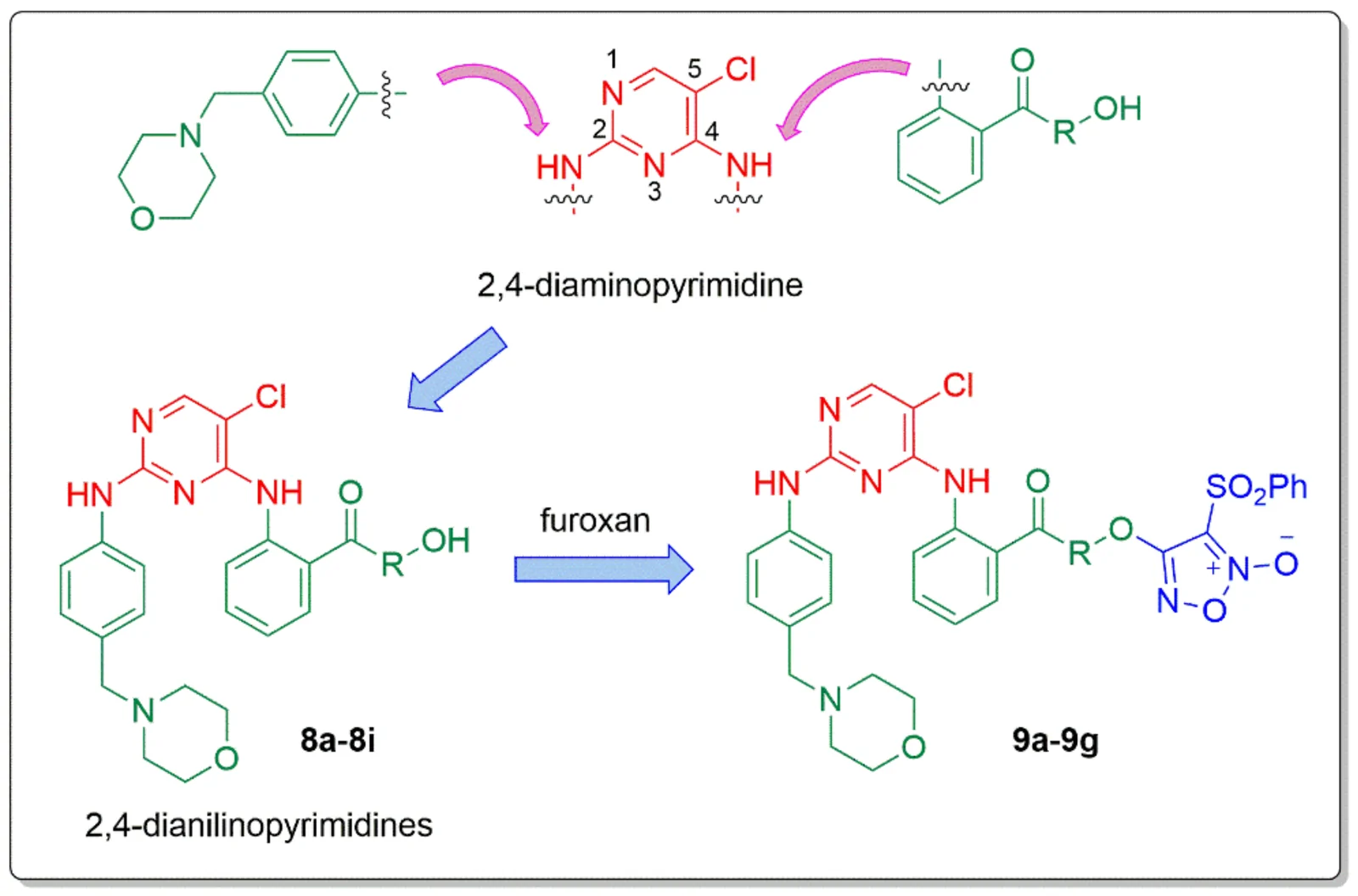
Design strategy of the target compounds.

With the aim of developing novel FAK-targeted inhibitors, a series of novel 2,4-dianilinopyrimidine derivatives containing 4-(morpholinomethyl)phenyl and *N*-substituted benzamides were designed and synthesized. In our design (Figure 2), we introduced a 4-(morpholinomethyl)phenyl moiety into the *N*^2^ position of the 2,4-diaminopyrimidine scaffold to provide flexibility and conformational freedom in order to better enable interactions with active site residues. In the *N*^4^ position of the scaffold, various alkanolamines were coupled with benzoic acid to form *N*-substituted benzamides to increase hydrogen bonding interactions with the target. Using this design approach, we synthesized and biologically evaluated the target 2,4-dianilinopyrimidines **8a**–**8i**. Moreover, nitric oxide (NO) plays an important role in cancer biology, and the use of NO donor-based therapies to fight cancer has generated significant progress [30]. Therefore, (phenylsulfonyl)furoxan was introduced into the scaffold as a potential NO donor, affording target compounds **9a**–**9g**. Their inhibitory activity against FAK, antiproliferative activity against tumor cells, cellular selectivity, apoptosis-inducing activities, and effects on cell cycle distribution and cell migration were thoroughly evaluated. In addition, molecular docking studies were also performed to elucidate the binding interactions between the inhibitor and FAK.

## 2. Results and Discussion

### 2.1. Synthetic Pathways

**Scheme 1:**
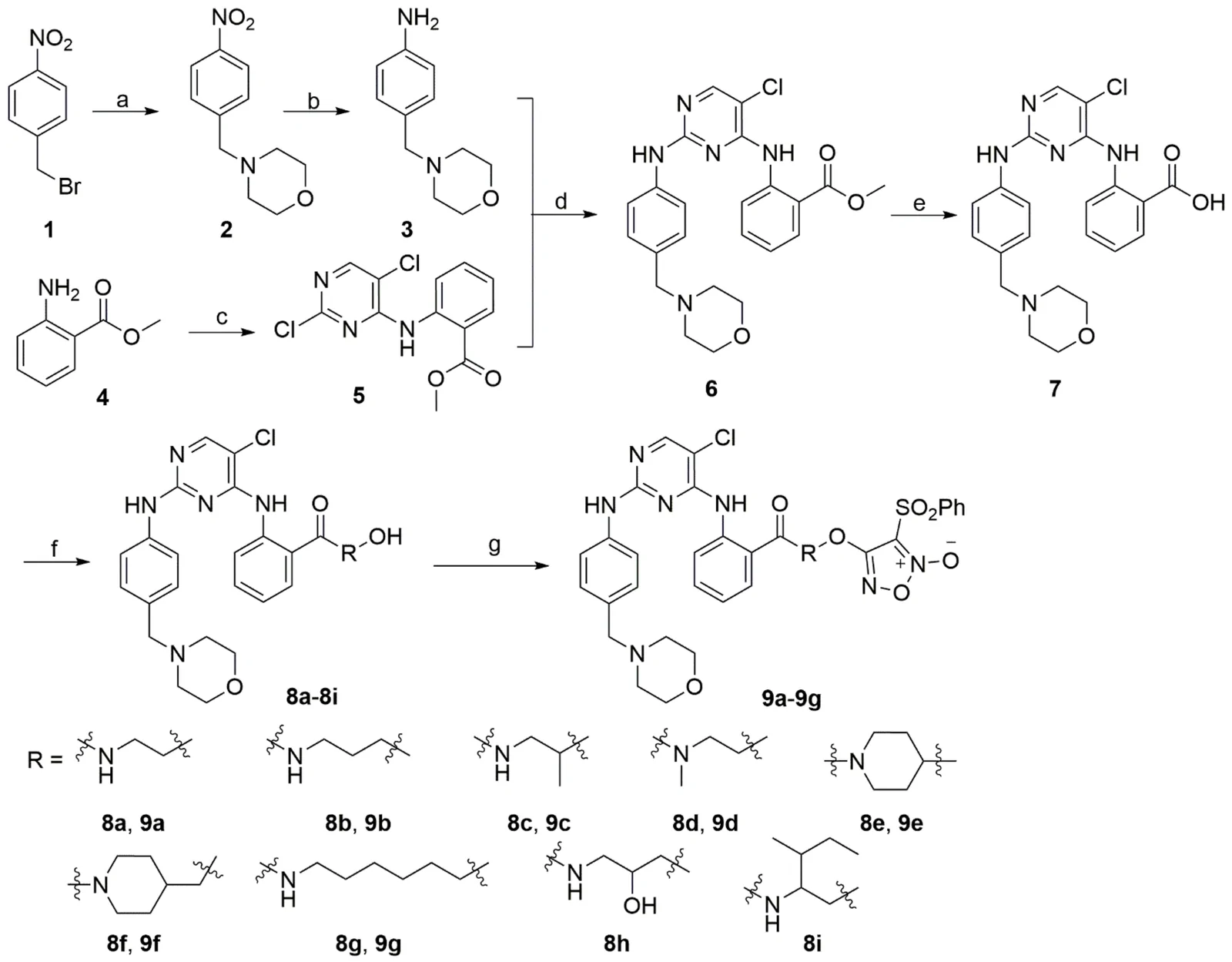
Synthesis of 2,4-dianilinopyrimidines derivatives **8a**–**8i** and **9a**–**9g**. Reagents and conditions: (**a**) morpholine, K_2_CO_3_, DMSO, 90 °C, 3 h, 94%. (**b**) 10% Pd/C, EtOH, H_2_ atm, rt, 8 h, 89%. (**c**) 2,4,5-trichloropyrimidine, DIPEA, 2-BuOH, 80 °C, 72 h, 76%. (**d**) TFA, 2-BuOH, 90 °C, 48 h, 25%. (**e**) NaOH, 1,4-dioxane/H_2_O, rt, 5 h, 95%. (**f**) HROH, EDCI, HOBt, DMF, rt, 24 h. (**g**) 3,4-bis(phenylsulfonyl)-1,2,5-oxadiazole-2-oxide (**10**), NaH, THF, 0 °C, 24 h.

The synthesis of compounds **8a**–**8i** and **9a**–**9g** is illustrated in Scheme 1. The substituted aniline **3** was prepared from 4-nitrobenzyl bromide (**1**) by amination with morpholine and subsequent reduction of the nitro group. Meanwhile, anilinopyrimidine **5** was prepared by nucleophilic aromatic substitution of the 4-Cl of 2,4,5-trichloropyrimidine with the nucleophilic aniline **4**. Then, aniline **3** underwent a regioselective nucleophilic aromatic substitution with the 2-Cl of **5** to obtain the 2,4-dianilinopyrimidines **6**. After saponification, intermediate **6** was converted to the corresponding acid **7**, which was condensed with various alkanolamines to yield the target compounds **8a**–**8i**. Compounds **8a**–**8g** were then reacted with 3,4-bis(phenylsulfonyl)-1,2,5-oxadiazole-2-oxide (**10**) to afford the target compounds **9a**–**9g**.

### 2.2. Kinase Inhibitory Activity and Structure–Activity Relationships

The inhibitory activity of the target compounds **8a**–**8i** and **9a**–**9g** against FAK kinase was evaluated using the ADP-Glo^TM^ assay, with TAE226 employed as the positive control. As provided in Table 1, most of these novel derivatives suppressed FAK activity at low-nanomolar concentrations. Compounds **8a**–**8g** featuring hydroxyl groups exhibited higher potencies compared to the corresponding furoxan-containing derivatives **9a**–**9g**. In particular, **8a** (IC_50_ = 0.047 ± 0.006 μM), **8c** (IC_50_ = 0.030 ± 0.007 μM), and **8d** (IC_50_ = 0.040 ± 0.011 μM) displayed the highest inhibitory activities against FAK compared to the other compounds tested. In addition, compounds **8c**, **8d**, **9c** (IC_50_ = 0.089 ± 0.014 μM), and **9d** (IC_50_ = 0.066 ± 0.014 μM), which featured methyl substituents at R, had higher inhibitory constants against FAK compared to **8i** (IC_50_ = 0.126 ± 0.021 μM), which featured a bulkier group for R. However, the presence of an additional hydroxyl group of R in **8h** (IC_50_ = 0.072 ± 0.007 μM) did not furnish an increase in the inhibition against FAK relative to **8b** (IC_50_ = 0.061 ± 0.009 μM). Lastly, analogues **8e**–**8g** featuring R with cyclic or longer alkanolamines exhibited lower inhibitory constants against FAK compared to compounds **8a**, **8c**, and **8d**.

### 2.3. Antiproliferative Activity against Tumor Cells

We next determined the antiproliferative effects of the 2,4-dianilinopyrimidines derivatives in H1975 non-small cell lung cancer (NSCLC) cells and A431 human epithelial carcinoma cells using a CCK-8 assay, with TAE226 and osimertinib (AZD9291, a potent EGFR inhibitor against the H1975 cell line [31]) employed as controls. As shown in Table 1, most of the target compounds were potent in the inhibition of H1975 and A431 cells. Consistent with the trends of their kinase inhibitory activities, compounds **8a**–**8g** displayed better inhibitory effects than **9a**–**9g** against the two tumor cell lines. In general, most compounds exhibited stronger inhibitory activity against H1975 cells than A431 cells. In addition, **8a** (IC_50_ = 0.044 ± 0.011 μM), **8b** (IC_50_ = 0.075 ± 0.011 μM), and **9a** (IC_50_ = 0.047 ± 0.009 μM) demonstrated higher antiproliferative activities against H1975 cells, while nine compounds (**8a**–**8f**, **8h**, **9a**, and **9b**) were found to more potently inhibit the proliferation of A431 cells, with IC_50_ values of 0.047–0.238 µM, than the positive controls. Combining these results, compounds **8a**, **8b**, and **9a** potently inhibit the proliferation of both H1975 and A431 cell lines.

### 2.4. Cellular Selectivity Assay

**Figure 3:**
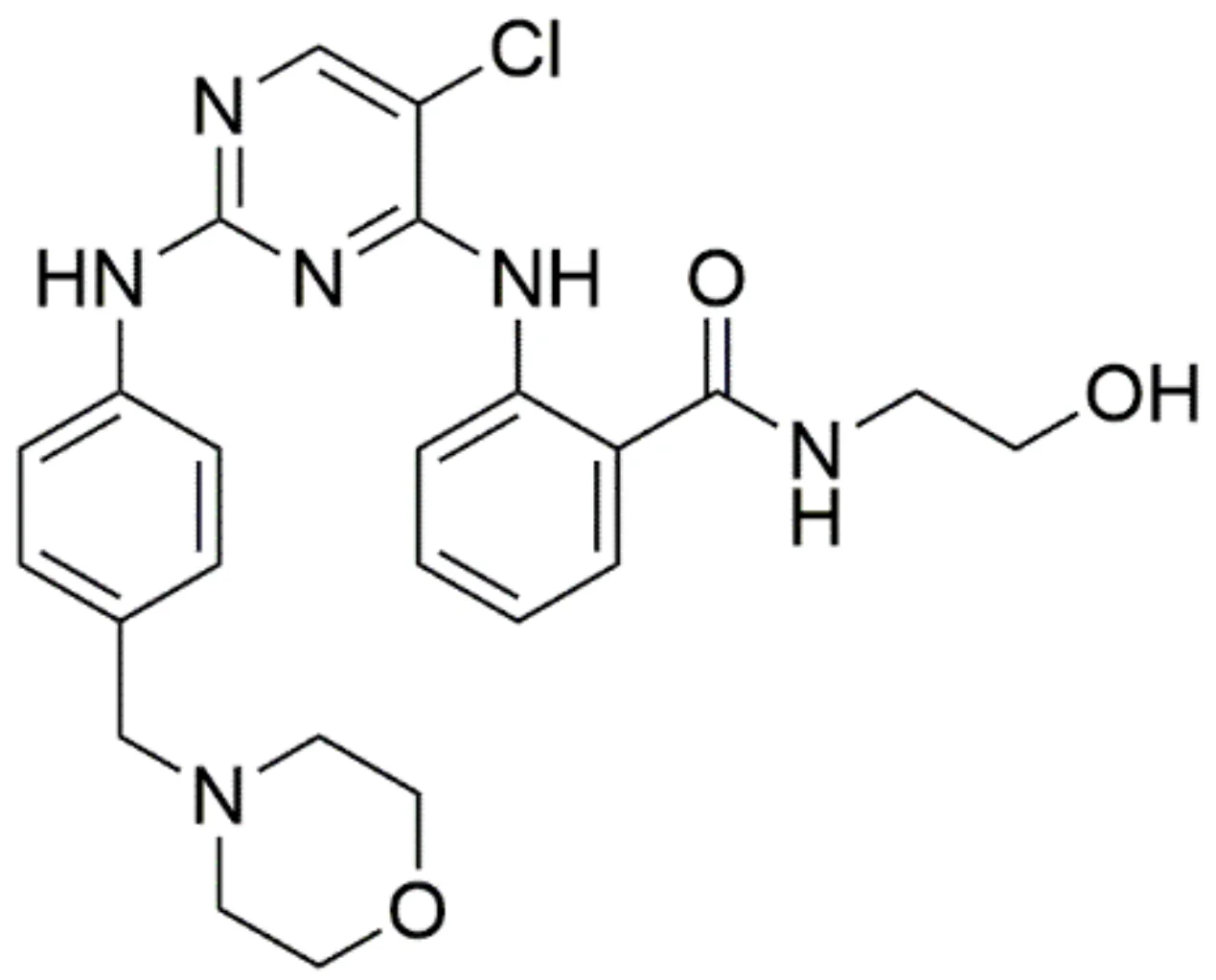
Structure of compound **8a**.

To determine the selectivity of the target compounds against tumor cells, normal human bronchial epithelial cells (HBE) were exposed to each of the compounds, and the viability of the cells was assessed using the CCK-8 assay. From these results, the selectivity index (SI) of the compounds between HBE and H1975 cells was also calculated. As shown in Table 1, most of these compounds more weakly inhibited the growth of HBE cells compared to H1975 cells. The SI values of the three most active compounds previously identified (**8a**, **8b**, and **9a**) were 24, 15, and 19, respectively. Since compound **8a** (Figure 3) displayed the best combination between potency and SI of the rest of the compounds evaluated, it was carried forward for further studies.

### 2.5. Cell Apoptosis Assay

**Figure 4:**
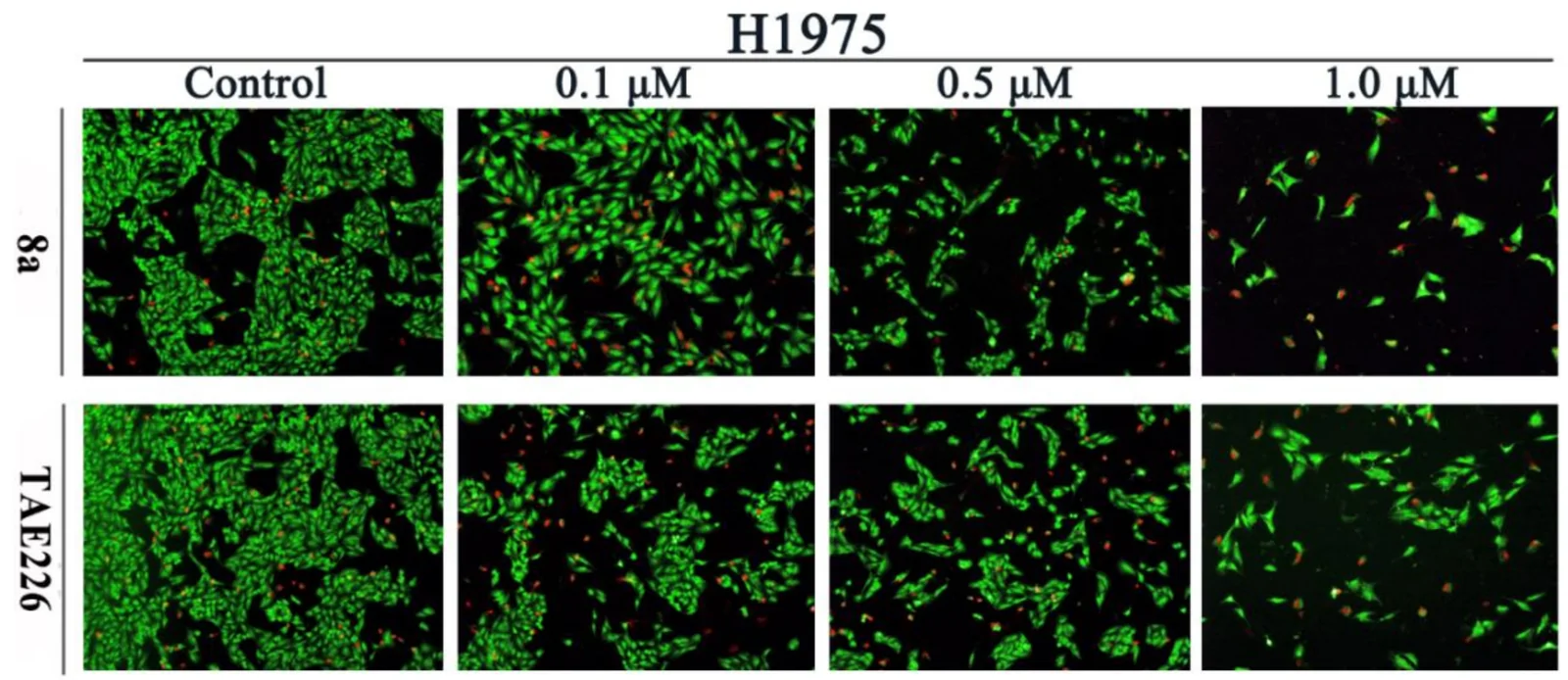
Apoptotic morphology of H1975 cells was assessed by AO/EB double fluorescent staining (100×, final magnification).

**Figure 5:**
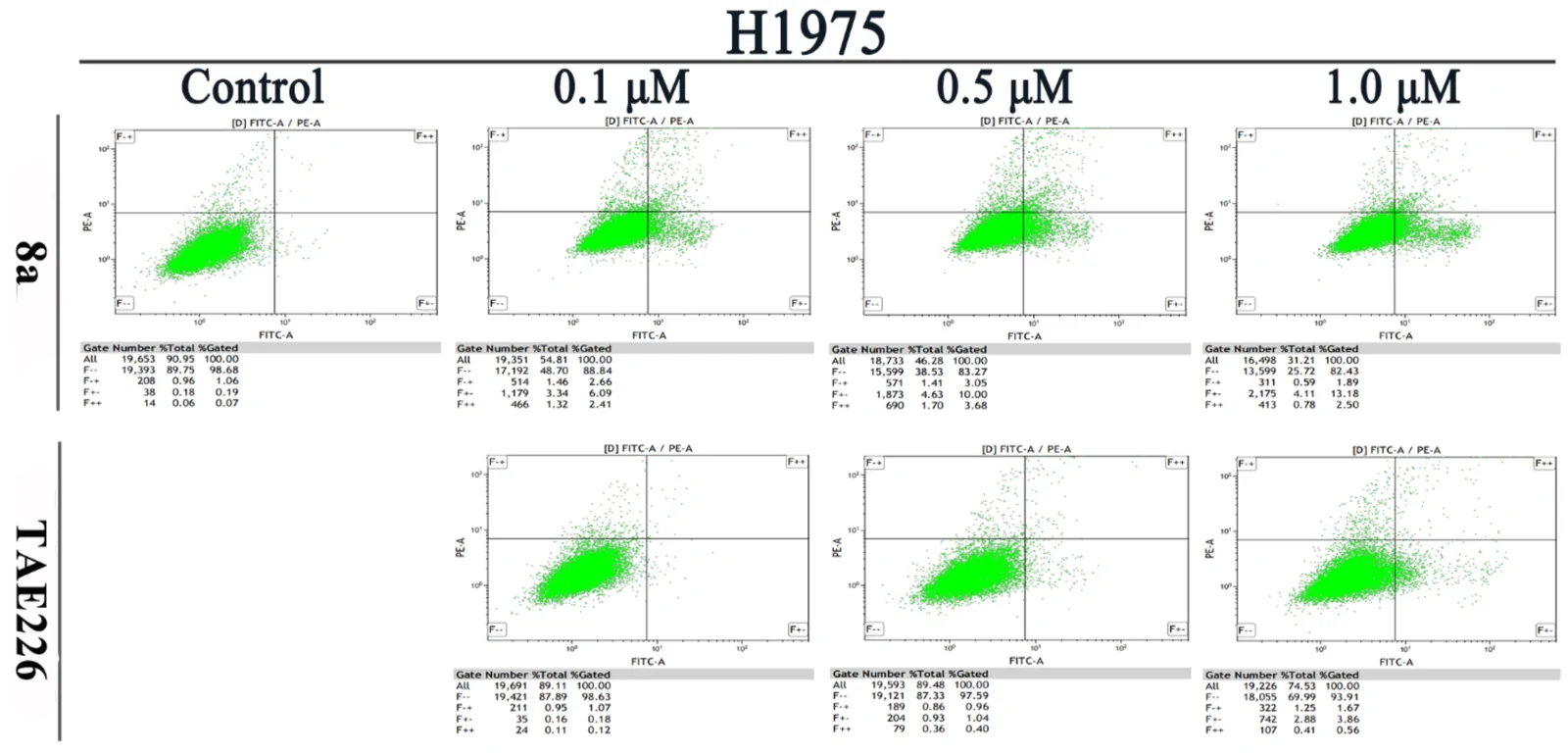
Flow cytometry analysis of apoptosis induction in H1975 cells treated with compound **8a** and TAE-226 (0.1, 0.5, 1 μM) for 48 h, and untreated cells (control) as a control. The cells were stained with Annexin V-FITC and PI, followed by flow cytometry analysis.

**Figure 6:**
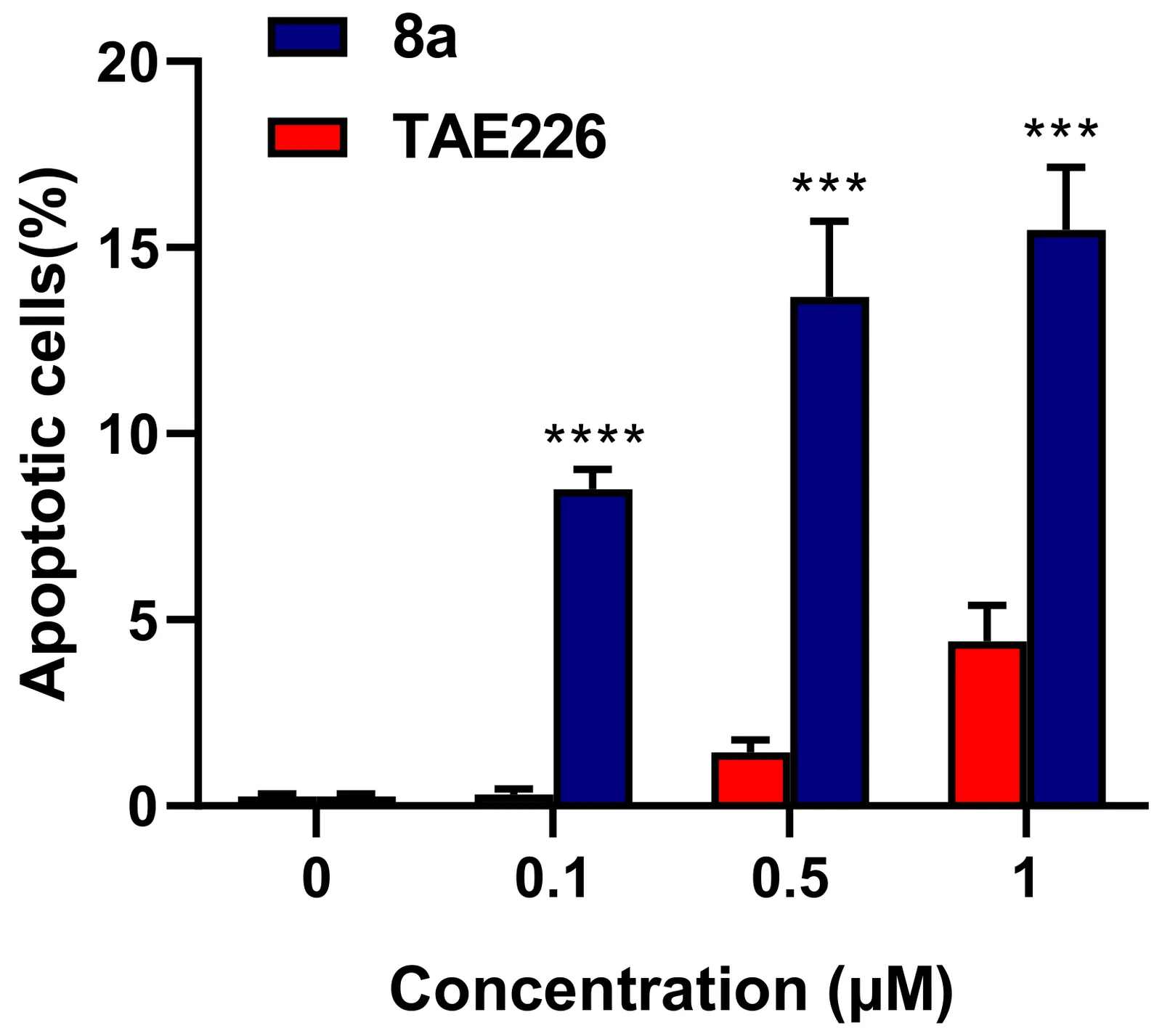
The quantitative data of the apoptosis of compound **8a** and TAE226 are expressed as the mean ± SD of the percentages of apoptotic cells from three independent experiments. Key: (***) *p* < 0.001, (****) *p* < 0.0001 vs. TAE226 group.

To better elucidate the mechanism by which these compounds inhibit cellular proliferation, AO/EB staining assays and flow cytometry were performed in H1975 cells to assess the effects of compound **8a** on inducing cellular apoptosis, using TAE226 as a positive control. As illustrated in Figure 4, H1975 cells that were treated with increasing concentrations (0.1, 0.5, and 1 μM) of **8a** exhibited certain morphological phenomena characteristic of apoptosis, including noticeable nuclear condensation, membrane blebbing, nuclear fragmentation, and the formation of apoptotic bodies. In addition, the results of flow cytometric analysis with Annexin V-FITC/PI double staining are summarized in Figure 5 and Figure 6. It was found that **8a** substantially increased the extent of apoptosis in H1975 cells in a dose-dependent manner. Notably, the ability of **8a** to induce apoptosis was significantly stronger compared to the control TAE226.

### 2.6. Cell Cycle Analysis

**Figure 7:**
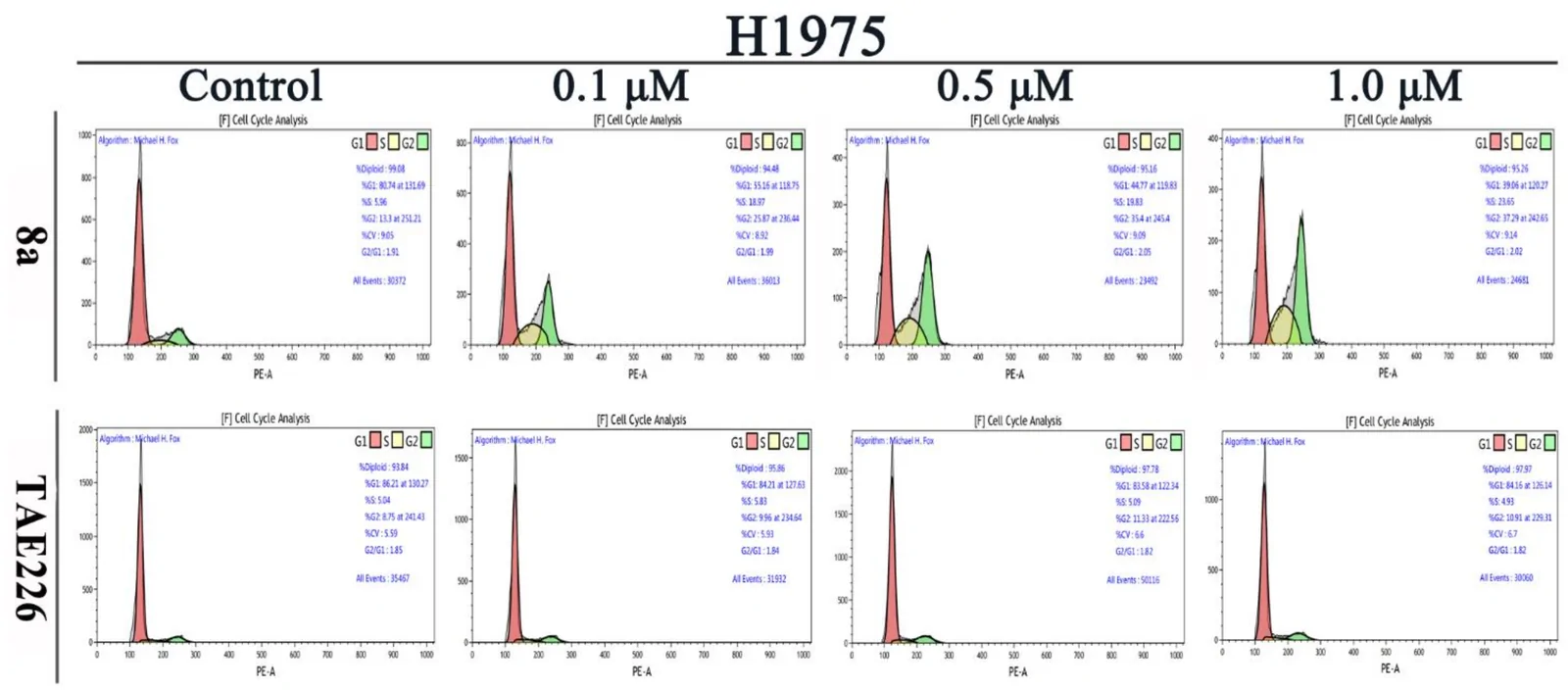
Cell cycle analysis of H1975 cells treated with **8a** and TAE226 (0.1, 0.5, 1 μM) for 48 h by flow cytometry assay.

**Figure 8:**
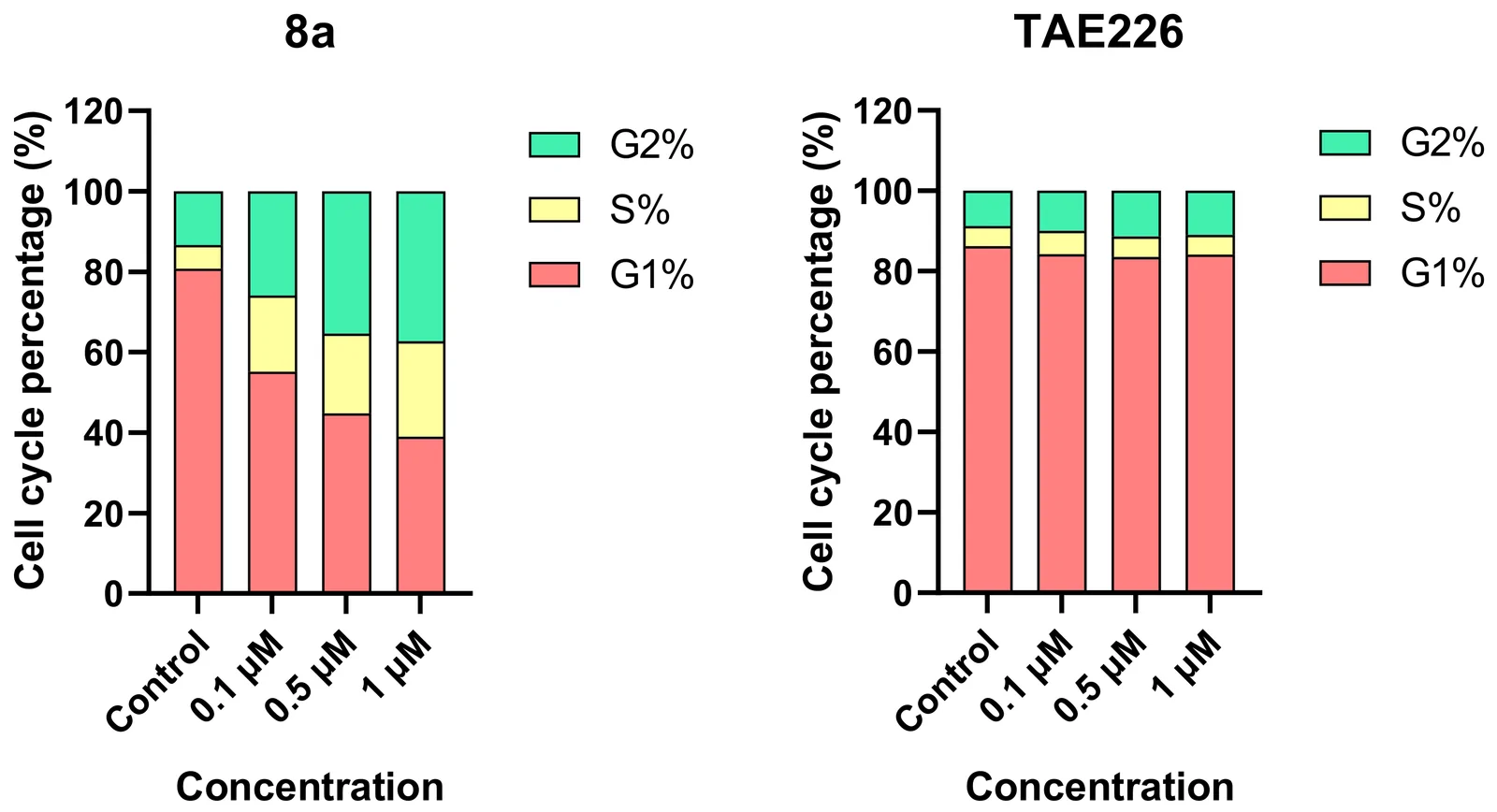
The cell cycle percentage of H1975 cells treated with **8a** and TAE226.

The effects of compound **8a** on cell cycle distribution in H1975 cells were analyzed by flow cytometry, and TAE226 was employed as a reference drug (Figure 7 and Figure 8). When the H1975 cells were exposed to compound **8a** at concentrations of 0.1, 0.5, and 1 μM for 48 h, the percentages of cells in G0/G1 phase decreased from 80.74% to 39.06%, while those in S phase and G2/M phase increased from 5.96% to 23.65% and 13.3% to 37.29% respectively, compared to the control group. Furthermore, treatment of the H1975 cells with compound **8a** induced cell cycle arrest in S/G2 phase in a dose-dependent manner, which was more potent than TAE226.

### 2.7. Cell Migration Assay

**Figure 9:**
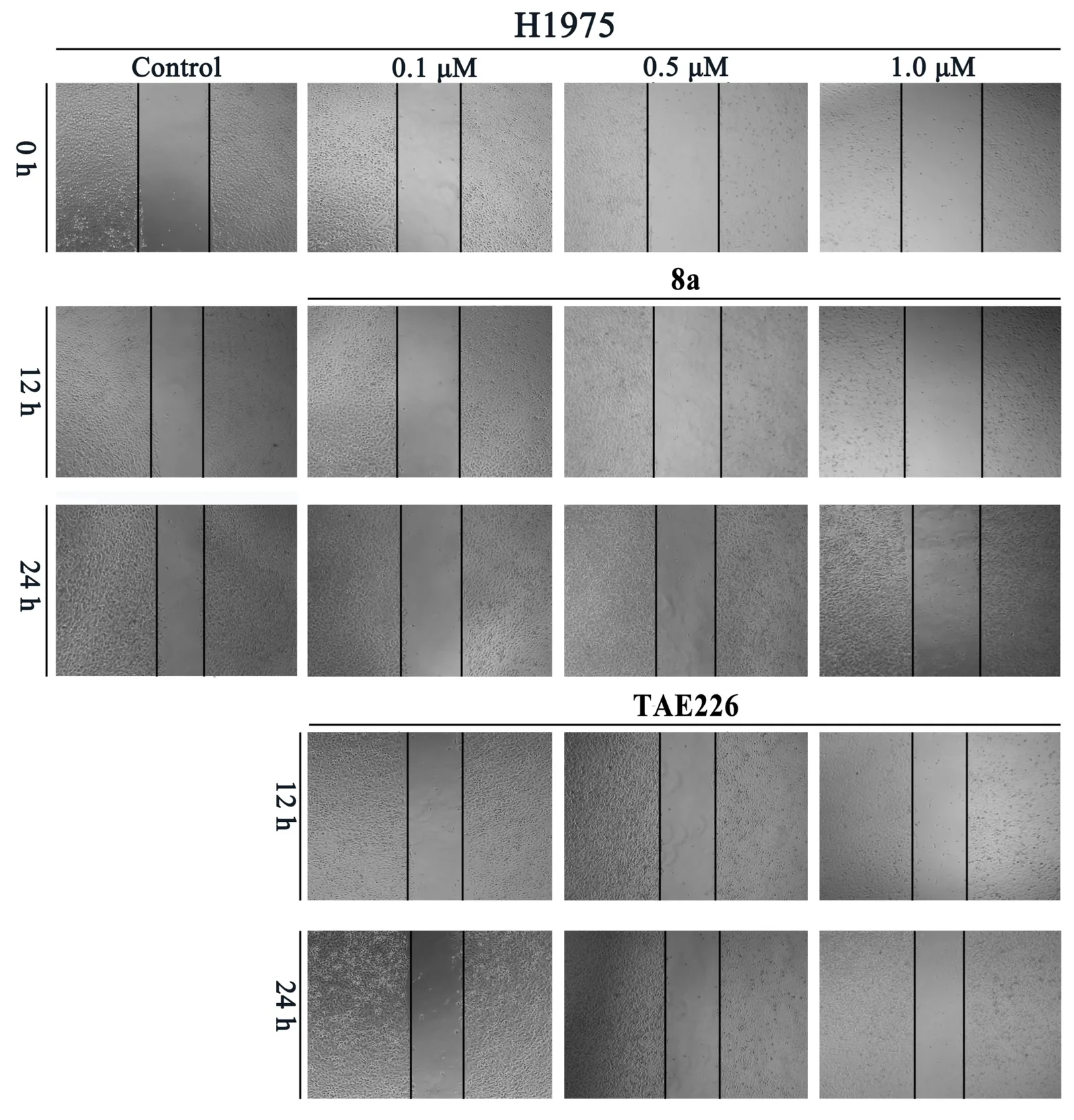
Effects of compound **8a** and TAE226 on the migration of H1975 cells in vitro determined by the scratch assay. H1975 cells were incubated with different concentrations of compound **8a** and TAE226 (0.1, 0.5, and 1 μM) for 24 h.

We further assessed whether compound **8a** had any influence on inhibiting the migration of H1975 cells using a scratch assay (Figure 9). H1975 cells were incubated with different concentrations of compound **8a** and TAE226 (0.1, 0.5, and 1 μM) for 24 h. As expected, H1975 cells in control groups displayed the farthest cell migration, whereas incubation of the cells with either compound **8a** or TAE226 manifested reduced migration distances of the H1975 cells. Furthermore, compound **8a** inhibited the migration of the H1975 cells slightly more potently than TAE226.

### 2.8. Molecular Docking Study

**Figure 10:**
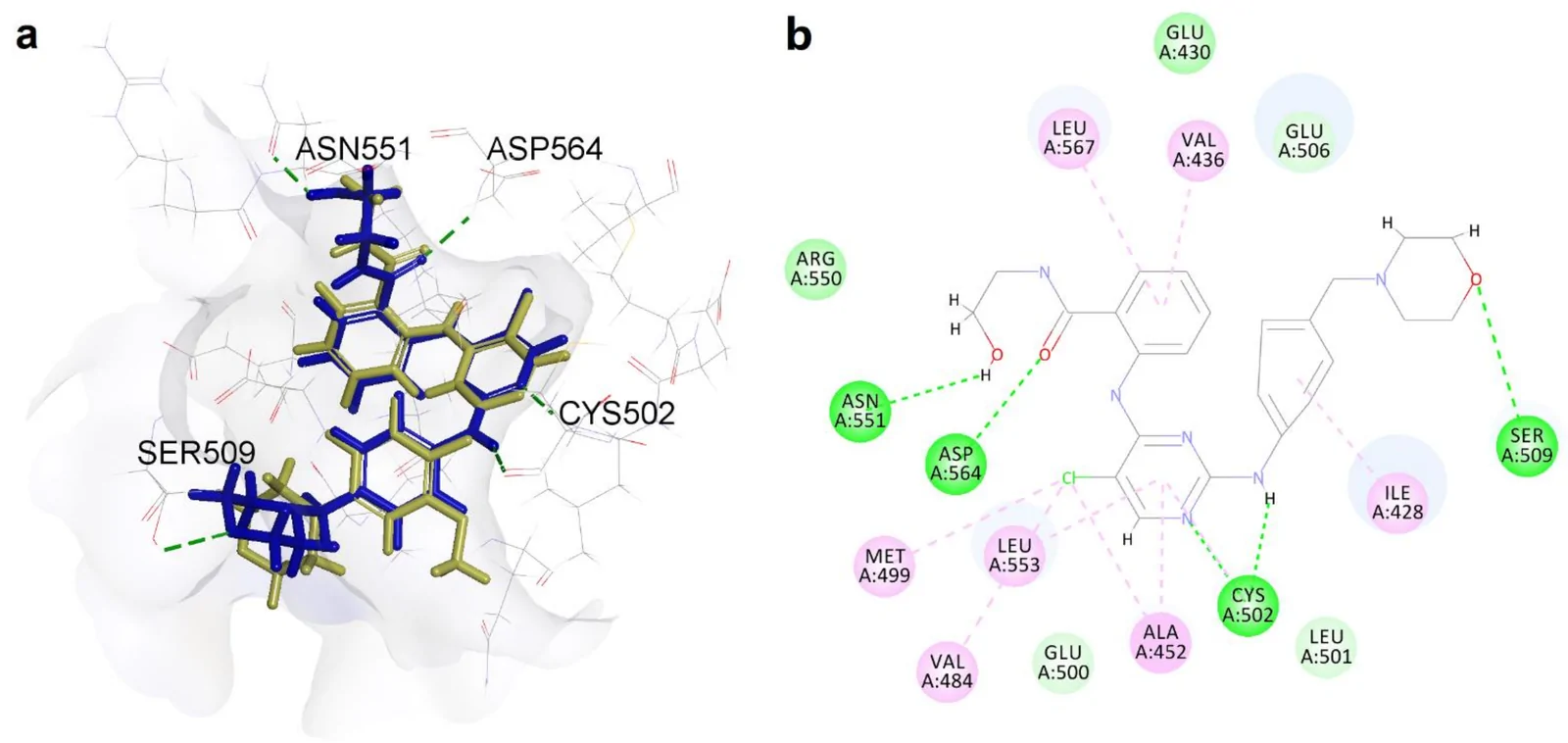
The predicted docked pose of compound **8a** in the FAK active site (PDB: 2JKK). (**a**) Image showing the overlap of compound **8a** (blue sticks) and TAE226 (yellow sticks). Hydrogen bonds are shown in green dots. (**b**) Interactions between compound **8a** and the key residues in the bonding site, where different interactions are shown in different colors. Hydrogen bonds: green; hydrophobic interactions: pink.

Molecular docking studies were conducted using the CDOCKER module in the Discovery Studio 2017 R2 software to predict the binding affinity of compound **8a** in FAK (PDB: 2JKK [29]), and the docking results are shown in Figure 10. Compound **8a** was bound in the active pocket of FAK, and its binding pose was nearly overlapped with that of the ligand TAE226 that was co-crystallized with FAK. Two hydrogen bonds were formed between compound **8a** and FAK, one between the N-1 nitrogen of the pyrimidine ring of **8a** and the NH of the amino acid residue Cys502, and the other between the oxygen atom of the amide group and the NH of Asp564, which was also observed with TAE226. As expected, hydrogen bonds were also formed between the morpholine oxygen of compound **8a** and the OH of Ser509, as well as the terminal hydroxyl group and the oxygen atom of the carbonyl group of Asn551. Moreover, hydrophobic interactions between compound **8a** and the surrounding residues in the binding pocket were observed. These docking results provided vital structural information for further rational design of FAK inhibitors featuring this scaffold as well as derivative scaffolds.

## 3. Materials and Methods

### 3.1. Chemistry

Starting materials, reagents, and solvents were commercially available. Melting points of individual compounds were measured without correction on a SGWX-4 Micro Melting Point apparatus (Shanghai Precision & Scientific Instrument Co. Ltd., Shanghai, China). ^1^H and ^13^C Nuclear Magnetic Resonance (NMR) spectral data were recorded in DMSO-*d*_6_ on a Bruker 400′54 Ascend spectrometer (Bruker BioSpin AG, Faellanden, Switzerland) at 303 K with tetramethylsilane (TMS) as an internal standard. High-Resolution Mass Spectrometry (HRMS) determinations were obtained on a Bruker compact™ QTOF system (Bruker Daltonik GmbH, Bremen, Germany). Compound **3** was prepared from 4-nitrobenzyl bromide (**1**) by amination and subsequent reduction, as described previously [32]. 3,4-Bis(phenylsulfonyl)-1,2,5-oxadiazole-2-oxide (**10**) was prepared via a three-step reaction sequence using thiophenol as the starting material according to a previously described methodology [33].

#### 3.1.1. Methyl 2-((2,5-Dichloropyrimidin-4-yl)amino)benzoate (**5**)

2,4,5-Trichloropyrimidine (14.560 g, 0.0794 mol), methyl anthranilate (**4**, 10 g, 0.0662 mol), and *N*,*N*-diisopropylethylamine (DIPEA) (10.262 g, 0.0794 mol) were added to 100 mL of 2-BuOH. The reaction was heated at 80 °C for 3 days. After being cooled to room temperature, the precipitate was filtered out and washed with ethanol and water to obtain the desired product as a white solid (15 g, 76% yield). mp 230–232 °C. ^1^H NMR (400 MHz, DMSO-*d*_6_) *δ* 11.12 (s, 1H), 8.52 (s, 1H), 8.49 (d, *J* = 8.4 Hz, 1H), 8.03 (d, *J* = 7.1 Hz, 1H), 7.72 (t, *J* = 8.4 Hz, 1H), 7.27 (t, *J* = 7.6 Hz, 1H), 3.89 (s, 3H). ^13^C NMR (101 MHz, DMSO-*d*_6_) *δ* 168.21, 157.11, 156.94, 156.19, 139.79, 134.66, 131.36, 124.22, 122.09, 118.34, 115.39, 53.15. ESI-HRMS (*m/z*): [M + H]^+^ calcd for C_12_H_10_Cl_2_N_3_O_2_ 298.0150, obsd 298.0183.

#### 3.1.2. Methyl 2-((5-Chloro-2-((4-(morpholinomethyl)phenyl)amino)pyrimidin-4-yl)amino)benzoate (**6**)

A solution composed of intermediate **5** (7.753 g, 0.026 mol), **3** (5 g, 0.026 mol), and TFA (4.450 g, 0.039 mol) in 70 mL of 2-BuOH was stirred at 90 °C for 48 h. After being concentrated in vacuo, the residue was dissolved in a small amount of methanol and added dropwise into a saturated NaHCO_3_ aqueous solution. A yellow solid was precipitated and then dried after filtration. The product was purified by chromatography on silica gel, eluting with 120:1 (*v/v*) dichloromethane-methanol (3.64 g, 31% yield). mp 223–225 °C. ^1^H NMR (400 MHz, DMSO-*d*_6_) *δ* 10.97 (s, 1H), 9.50 (s, 1H), 8.91 (d, *J* = 8.5 Hz, 1H), 8.27 (s, 1H), 8.03 (d, *J* = 8.0 Hz, 1H), 7.63–7.57 (m, 3H), 7.30–7.09 (m, 3H), 3.91 (s, 3H), 3.64–3.51 (m, 4H), 3.41 (s, 2H), 2.35 (brs, 4H). ^13^C NMR (101 MHz, DMSO-*d*_6_) *δ* 168.47, 158.21, 155.40, 141.55, 139.37, 134.48, 131.67, 131.30, 129.54, 122.53, 121.59, 120.26, 115.99, 105.57, 66.66, 62.56, 53.56, 53.06. ESI-HRMS (*m/z*): [M + H]^+^ calcd for C_23_H_25_ClN_5_O_3_ 454.1640, obsd 454.1615.

#### 3.1.3. 2-((5-Chloro-2-((4-(morpholinomethyl)phenyl)amino)pyrimidin-4-yl)amino)benzoic acid (**7**)

NaOH (1.028 g, 25.7 mmol) was dissolved in 10 mL of water, which was added to a solution of compound **6** (2.913 g, 6.43 mmol) in 10 mL of 1,4-dioxane. The resulting mixture was stirred at room temperature for 5 h and acidified to subacidity (pH ≈ 6) with diluted hydrochloric acid. The precipitate was collected as a yellow sticky solid and then stirred in ethyl acetate to disperse. After filtration, a powdery yellow solid was obtained (2.7 g, 96% yield). mp > 300 °C. ^1^H NMR (400 MHz, DMSO-*d*_6_) *δ* 12.13 (s, 1H), 9.53 (s, 1H), 8.91 (d, *J* = 7.3 Hz, 1H), 8.19 (s, 1H), 8.05 (d, *J* = 7.5 Hz, 1H), 7.65 (d, *J* = 8.0 Hz, 2H), 7.49 (t, *J* = 7.2 Hz, 1H), 7.27 (d, *J* = 8.1 Hz, 2H), 7.09 (t, *J* = 7.3 Hz, 1H), 3.69 (t, *J* = 4.4 Hz, 6H), 2.64 (br s, 4H). ^13^C NMR (101 MHz, DMSO-*d*_6_) *δ* 170.60, 158.15, 155.52, 154.98, 141.72, 140.13, 133.29, 131.70, 130.20, 129.26, 122.06, 120.96, 120.03, 118.66, 105.92, 65.96, 61.80, 52.93. ESI-HRMS (*m/z*): [M + H]^+^ calcd for C_22_H_23_ClN_5_O_3_ 440.1484, obsd 440.1468.

#### 3.1.4. General Procedure for the Preparation of **8a**–**8i**

A flask was charged with compound **7** (200 mg, 0.455 mmol), EDCI (174 mg, 0.909 mmol), HOBt (80 mg, 0.591 mmol), and DMF (2 mL). After stirring for 0.5 h, alkanolamine (2.27 mmol) was added. The mixture continued to react overnight, and then was diluted with water (10 mL) and extracted thrice with ethyl acetate. The organic layers were combined, concentrated, and purified using flash chromatography (dichloromethane-methanol 20:1 *v/v*).

##### 2-((5-Chloro-2-((4-(morpholinomethyl)phenyl)amino)pyrimidin-4-yl)amino)-*N*-(2-hydroxyethyl)benzamide (**8a**)

The title compound was obtained starting from compound **7** and ethanolamine. As a yellow solid, 18% yield. mp 230–231 °C. ^1^H NMR (400 MHz, DMSO-*d*_6_) *δ* 11.55 (s, 1H), 9.47 (s, 1H), 8.82 (s, 1H), 8.75 (d, *J* = 6.7 Hz, 1H), 8.22 (s, 1H), 7.84 (d, *J* = 7.5 Hz, 1H), 7.62 (d, *J* = 7.7 Hz, 2H), 7.48 (t, *J* = 7.4 Hz, 1H), 7.20 (d, *J* = 8.0 Hz, 2H), 7.15 (t, *J* = 7.3 Hz, 1H), 4.83 (s, 1H), 3.57 (br s, 6H), 3.40 (s, 2H), 3.37 (t, *J* = 5.3 Hz, 2H), 2.34 (br s, 4H). ^13^C NMR (101 MHz, DMSO-*d*_6_) *δ* 169.10, 158.23, 155.44, 155.06, 139.74, 139.55, 131.86, 131.36, 129.55, 128.72, 122.36, 121.86, 121.41, 120.02, 105.48, 66.66, 62.58, 59.88, 53.57, 42.66. ESI-HRMS (*m/z*): [M + H]^+^ calcd for C_24_H_28_ClN_6_O_3_ 483.1906, obsd 483.1911.

##### 2-((5-Chloro-2-((4-(morpholinomethyl)phenyl)amino)pyrimidin-4-yl)amino)-*N*-(3-hydroxypropyl)benzamide (**8b**)

The title compound was obtained starting from compound **7** and 3-amino-1-propanol. As a yellow solid, 19% yield. mp 200–202 °C. ^1^H NMR (400 MHz, DMSO-*d*_6_) *δ* 11.48 (s, 1H), 9.44 (s, 1H), 8.82–8.67 (m, 2H), 8.22 (s, 1H), 7.77 (dd, *J* = 7.9, 1.3 Hz, 1H), 7.61 (d, *J* = 8.4 Hz, 2H), 7.47 (t, *J* = 8.4 Hz, 1H), 7.20 (d, *J* = 8.5 Hz, 2H), 7.16 (t, *J* = 7.6 Hz, 1H), 4.51 (t, *J* = 5.0 Hz, 1H), 3.58 (t, 4H, *J* = 4.4 Hz, 4H), 3.49 (q, *J* = 6.0 Hz, 2H), 3.41 (s, 2H), 3.38–3.35 (m, 2H), 2.35 (br s, 4H), 1.77–1.65 (quint, *J* = 6.0 Hz, 2H). ^13^C NMR (101 MHz, DMSO-*d*_6_) *δ* 168.92, 158.25, 155.45, 155.09, 139.65, 139.55, 131.80, 131.43, 129.55, 128.56, 122.44, 121.93, 121.66, 120.06, 105.46, 66.68, 62.59, 59.07, 53.58, 37.17, 32.60. ESI-HRMS (*m/z*): [M + H]^+^ calcd for C_25_H_30_ClN_6_O_3_ 497.2062, obsd 497.2062.

##### 2-((5-Chloro-2-((4-(morpholinomethyl)phenyl)amino)pyrimidin-4-yl)amino)-*N*-(2-hydroxypropyl)benzamide (**8c**)

The title compound was obtained starting from compound **7** and 1-amino-2-propanol. As a yellow solid, 23% yield. mp 232–233 °C. ^1^H NMR (400 MHz, DMSO-*d*_6_) *δ* 11.41 (s, 1H), 9.45 (s, 1H), 8.86–8.63 (m, 2H), 8.21 (s, 1H), 7.80 (d, *J* = 7.8 Hz, 1H), 7.60 (d, *J* = 8.1 Hz, 2H), 7.47 (t, *J* = 7.5 Hz, 1H), 7.19 (dd, *J* = 8.0 Hz, 2H), 7.16 (t, *J* = 7.2 Hz, 1H), 4.80 (d, *J* = 4.2 Hz, 1H), 3.81 (m, 1H), 3.57 (br s, 4H), 3.41 (s, 2H), 3.23 (t, *J* = 5.3 Hz, 2H), 2.35 (br s, 4H), 1.08 (d, *J* = 6.0 Hz, 3H). ^13^C NMR (101 MHz, DMSO-*d*_6_) *δ* 169.05, 158.27, 155.47, 155.08, 139.56, 131.78, 131.43, 130.10, 129.55, 128.72, 122.43, 121.94, 121.83, 120.08, 105.44, 66.68, 65.32, 62.57, 53.58, 47.53, 21.64. ESI-HRMS (*m/z*): [M + H]^+^ calcd for C_25_H_30_ClN_6_O_3_ 497.2062, obsd 497.2072.

##### 2-((5-Chloro-2-((4-(morpholinomethyl)phenyl)amino)pyrimidin-4-yl)amino)-*N*-(2-hydroxyethyl)-*N*-methylbenzamide (**8d**)

The title compound was obtained starting from compound **7** and 2-(methylamino)ethanol. As a yellow solid, 18% yield. mp 103–105 °C. ^1^H NMR (400 MHz, DMSO-*d*_6_) *δ* 9.35 (s, 1H), 9.05 (s, 1H), 8.16 (d, *J* = 7.8 Hz, 1H), 8.15 (s, 1H), 7.55 (d, *J* = 8.1 Hz, 2H), 7.50–7.38 (m, 2H), 7.24 (t, *J* = 7.6 Hz, 1H), 7.15–7.02 (m, 2H), 5.12 (br s, 1H), 3.56 (t, *J* = 4.0 Hz, 4H), 3.49 (q, *J* = 5.1 Hz, 2H), 3.41 (s, 2H), 3.34–3.28 (m, 2H), 2.97 (s, 3H), 2.32 (br s, 4H). ^13^C NMR (101 MHz, DMSO-*d*_6_) *δ* 169.95, 158.21, 156.15, 154.93, 139.64, 136.73, 131.08, 129.46, 128.15, 127.77, 124.74, 124.32, 123.88, 119.52, 104.63, 66.66, 62.56, 57.60, 53.56, 50.08, 38.96. ESI-HRMS (*m/z*): [M + H]^+^ calcd for C_25_H_30_ClN_6_O_3_ 497.2062, obsd 497.2065.

##### 2-((5-Chloro-2-((4-(morpholinomethyl)phenyl)amino)pyrimidin-4-yl)amino)phenyl)(4-hydroxypiperidin-1-yl)methanone (**8e**)

The title compound was obtained starting from compound **7** and 4-hydroxypiperidine. As a yellow solid, 20% yield. mp 138–140 °C. ^1^H NMR (400 MHz, DMSO-*d*_6_) *δ* 9.36 (s, 1H), 8.89 (s, 1H), 8.17 (s, 1H), 8.14 (d, *J* = 8.2 Hz, 1H), 7.52 (d, *J* = 8.4 Hz, 2H), 7.47 (t, *J* = 7.2 Hz, 1H), 7.37 (d, *J* = 7.6 Hz, 1H), 7.26 (t, *J* = 7.4 Hz, 1H), 7.09 (d, *J* = 8.4 Hz, 2H), 4.77 (d, *J* = 3.4 Hz, 1H), 4.00 (br s, 1H), 3.72–3.62 (m, 1H), 3.56 (t, *J* = 4.0 Hz, 4H), 3.48 (br s, 1H), 3.37 (s, 2H), 3.23 (br s, 1H), 3.09 (br s, 1H), 2.32 (br s, 4H), 1.76 (br s, 1H), 1.52 (br s, 1H), 1.35 (br s, 1H), 1.15 (br s, 1H). ^13^C NMR (101 MHz, DMSO-*d*_6_) *δ* 168.01, 158.20, 156.07, 155.04, 139.60, 136.52, 131.06, 130.17, 129.44, 128.53, 127.83, 125.25, 124.40, 119.57, 104.44, 66.66, 65.69, 62.55, 53.55, 45.23, 34.95. ESI-HRMS (*m/z*): [M + H]^+^ calcd for C_27_H_32_ClN_6_O_3_ 523.2219, obsd 523.2213.

##### 2-((5-Chloro-2-((4-(morpholinomethyl)phenyl)amino)pyrimidin-4-yl)amino)phenyl)(4-(hydroxymethyl)piperidin-1-yl)methanone (**8f**)

The title compound was obtained starting from compound **7** and 4-(hydroxymethyl)piperidine. As a yellow solid, 11% yield. mp 92–94 °C. ^1^H NMR (400 MHz, DMSO-*d*_6_) *δ* 9.40 (s, 1H), 8.90 (s, 1H), 8.20 (s, 1H), 8.18 (d, *J* = 8.2 Hz, 1H), 7.62–7.45 (m, 3H), 7.37 (d, *J* = 7.6 Hz, 1H), 7.27 (t, *J* = 7.4 Hz, 1H), 7.12 (d, *J* = 7.6 Hz, 2H), 4.51 (br s, 2H), 3.58 (brs, 5H), 3.41 (s, 2H), 3.20 (br s, 2H), 2.97(br s, 1H), 2.75(br s, 1H), 2.32 (br s, 4H), 2.12–1.85 (m, 1H), 1.84–1.41(m, 4H). ^13^C NMR (101 MHz, DMSO-*d*_6_) *δ* 167.98, 158.20, 155.99, 155.03, 139.60, 136.49, 131.04, 130.13, 129.46, 128.44, 127.80, 125.09, 124.32, 119.56, 104.46, 66.65, 65.91, 62.56, 53.55, 47.80, 41.99, 38.73, 29.51, 28.87. ESI-HRMS (*m/z*): [M + H]^+^ calcd for C_28_H_34_ClN_6_O_3_ 537.2375, obsd 537.2362.

##### 2-((5-Chloro-2-((4-(morpholinomethyl)phenyl)amino)pyrimidin-4-yl)amino)-*N*-(6-hydroxyhexyl)benzamide (**8g**)

The title compound was obtained starting from compound **7** and 6-amino-1-hexanol. As a yellow solid, 17% yield. mp 197–198 °C. ^1^H NMR (400 MHz, DMSO-*d*_6_) *δ* 11.42 (s, 1H), 9.43 (s, 1H), 8.74 (t, *J* = 5.5 Hz, 1H), 8.70 (d, *J* = 8.0 Hz, 1H), 8.21 (s, 1H), 7.75 (d, *J* = 7.6 Hz, 1H), 7.60 (d, *J* = 7.9 Hz, 2H), 7.47 (t, *J* = 7.5 Hz, 1H), 7.20 (d, *J* = 8.1 Hz, 2H), 7.16 (t, *J* = 7.5 Hz, 1H), 4.34 (t, *J* = 4.4 Hz, 1H), 3.58 (t, *J* = 4.0 Hz, 4H), 3.44–3.39 (m, 4H), 3.28 (q, *J* = 5.6 Hz, 2H), 2.36 (br s, 4H), 1.54 (quint, *J* = 6.0 Hz, 2H), 1.42 (quint, *J* = 6.0 Hz, 2H), 1.36–1.28 (m, 4H). ^13^C NMR (101 MHz, DMSO-*d*_6_) *δ* 168.84, 158.26, 155.49, 155.07, 139.38, 131.73, 131.41, 130.11, 129.59, 128.53, 122.48, 121.95, 120.07, 105.47, 66.64, 62.55, 61.14, 53.55, 32.95, 29.38, 26.86, 25.75. ESI-HRMS (*m/z*): [M + H]^+^ calcd for C_28_H_36_ClN_6_O_3_ 539.2532, obsd 539.2521.

##### 2-((5-Chloro-2-((4-(morpholinomethyl)phenyl)amino)pyrimidin-4-yl)amino)-*N*-(2,3-dihydroxypropyl)benzamide (**8h**)

The title compound was obtained starting from compound **7** and 3-amino-1,2-propanediol. As a yellow solid, 15% yield. mp 159–161 °C. ^1^H NMR (400 MHz, DMSO-*d*_6_) *δ* 11.46 (s, 1H), 9.46 (s, 1H), 8.78–8.66 (m, 2H), 8.22 (s, 1H), 7.81 (d, *J* = 7.9 Hz, 1H), 7.61 (d, *J* = 8.4 Hz, 2H), 7.48 (t, *J* = 7.8 Hz, 1H), 7.20 (t, *J* = 8.6 Hz, 2H), 7.15 (d, *J* = 7.5 Hz, 1H), 4.87 (d, *J* = 4.7 Hz, 1H), 4.62 (t, *J* = 6.0 Hz, 1H), 3.73–3.65 (m, 1H), 3.57 (t, *J* = 4.4 Hz, 4H), 3.46–3.42 (m, 1H), 3.41 (s, 2H), 3.40–3.37 (m, 2H), 3.27–3.16 (m, 1H), 2.35 (br s, 4H). ^13^C NMR (101 MHz, DMSO-*d*_6_) *δ* 169.14, 158.23, 155.43, 155.09, 139.63, 139.55, 131.84, 131.36, 129.57, 128.75, 122.41, 121.89, 121.61, 120.03, 105.46, 70.48, 66.66, 64.51, 62.57, 53.57, 43.51. ESI-HRMS (*m/z*): [M + H]^+^ calcd for C_25_H_30_ClN_6_O_4_ 513.2012, obsd 513.1995.

##### 2-((5-Chloro-2-((4-(morpholinomethyl)phenyl)amino)pyrimidin-4-yl)amino)-*N*-(1-hydroxy-3-methylpentan-2-yl)benzamide (**8i**)

The title compound was obtained starting from compound **7** and L-isoleucinol. As a yellow solid, 20% yield. mp 264–265 °C. ^1^H NMR (400 MHz, DMSO-*d*_6_) *δ* 11.27 (s, 1H), 9.46 (s, 1H), 8.67 (d, *J* = 7.1 Hz, 1H), 8.37 (d, *J* = 8.6 Hz, 1H), 8.21 (s, 1H), 7.81 (d, *J* = 7.7 Hz, 1H), 7.61 (d, *J* = 8.0 Hz, 2H), 7.47 (t, *J* = 7.6 Hz, 1H), 7.22–7.14 (m, 3H), 4.61 (t, *J* = 5.1 Hz, 1H), 3.94–3.84 (m, 1H), 3.62–3.49 (m, 6H), 3.41 (s, 2H), 2.35 (br s, 4H), 1.78–1.65 (m, 1H), 1.52–1.42 (m, 1H), 1.19–1.09 (m, 1H), 0.90 (d, *J* = 6.6 Hz, 3H), 0.85 (t, *J* = 7.2 Hz, 3H). ^13^C NMR (101 MHz, DMSO-*d*_6_) *δ* 168.87, 158.26, 155.49, 155.06, 139.58, 139.28, 131.57, 131.37, 129.57, 128.90, 122.70, 122.45, 121.96, 120.04, 105.38, 66.65, 62.56, 61.42, 56.05, 53.55, 35.44, 25.49, 16.04, 11.59. ESI-HRMS (*m/z*): [M + H]^+^ calcd for C_28_H_36_ClN_6_O_3_ 539.2532, obsd 539.2528.

#### 3.1.5. General Procedure for the Preparation of **9a**–**9g**

To a solution of compounds **8a**–**8g** (0.195 mmol) in 2 mL of THF, NaH (14 mg, 0.585 mmol) was slowly added under stirring at 0 °C. After stirring for 5 min, 3,4-bis(phenylsulfonyl)-1,2,5-oxadiazole-2-oxide (71.4 mg, 0.195 mmol) was added, and the obtained mixture was allowed to react overnight at room temperature, then quenched by a few drops of water and concentrated in vacuo. The crude product was purified by column chromatography (dichloromethane-methanol 40:1 *v/v*).

##### 4-(2-(2-((5-Chloro-2-((4-(morpholinomethyl)phenyl)amino)pyrimidin-4-yl)amino)benzamido)ethoxy)-3-(phenylsulfonyl)-1,2,5-oxadiazole 2-oxide (**9a**)

As a yellow solid, 15% yield. mp 174–176 °C. ^1^H NMR (400 MHz, DMSO-*d*_6_) *δ* 11.48 (s, 1H), 9.49 (s, 1H), 9.11 (s, 1H), 8.79 (d, *J* = 6.0 Hz, 1H), 8.21 (s, 1H), 7.97 (d, *J* = 6.9 Hz, 2H), 7.86 (d, *J* = 6.7 Hz, 1H), 7.75 (d, *J* = 6.5 Hz, 1H), 7.62 (d, *J* = 6.7 Hz, 2H), 7.60–7.47 (m, 3H), 7.21 (d, *J* = 6.2 Hz, 3H), 4.61 (br s, 2H), 3.78 (br s, 2H), 3.58 (br s, 4H), 3.44 (s, 2H), 2.37 (br s, 4H). ^13^C NMR (101 MHz, DMSO-*d*_6_) *δ* 169.44, 159.34, 158.19, 155.42, 155.07, 139.97, 139.63, 137.68, 136.43, 132.23, 130.29, 130.10, 129.64, 128.75, 122.39, 121.95, 120.85, 120.02, 110.99, 105.56, 69.84, 66.57, 62.47, 53.49, 38.40. ESI-HRMS (*m/z*): [M + H]^+^ calcd for C_32_H_32_ClN_8_O_7_S 707.1798, obsd 707.1798.

##### 4-(3-(2-((5-Chloro-2-((4-(morpholinomethyl)phenyl)amino)pyrimidin-4-yl)amino)benzamido)propoxy)-3-(phenylsulfonyl)-1,2,5-oxadiazole 2-oxide (**9b**)

As a yellow solid, 17% yield. mp 144–146 °C. ^1^H NMR (400 MHz, DMSO-*d*_6_) *δ* 11.40 (s, 1H), 9.44 (s, 1H), 8.87 (t, *J* = 5.7 Hz, 1H), 8.71 (d, *J* = 8.1 Hz, 1H), 8.19 (s, 1H), 8.06 (dd, *J* = 8.4, 1.1 Hz, 2H), 7.90 (t, *J* = 7.6 Hz, 1H), 7.80 (dd, *J* = 7.9, 1.3 Hz, 1H), 7.76 (t, *J* = 7.9 Hz, 2H), 7.61 (d, *J* = 8.4 Hz, 2H), 7.49 (t, *J* = 7.8 Hz, 1H), 7.22–7.15 (m, 3H), 4.47 (t, *J* = 6.0 Hz, 2H), 3.58 (t, *J* = 4.4 Hz, 4H), 3.47 (q, *J* = 6.3 Hz, 2H), 3.42 (s, 2H), 2.36 (br s, 4H), 2.12–2.01 (quint, *J* = 6.0 Hz, 2H). ^13^C NMR (101 MHz, DMSO-*d*_6_) *δ* 169.21, 159.40, 158.23, 155.43, 155.06, 139.67, 139.57, 137.65, 136.60, 131.93, 130.53, 129.58, 128.87, 128.63, 122.46, 121.98, 121.54, 120.06, 110.97, 105.45, 69.61, 66.64, 62.54, 53.55, 36.02, 28.61. ESI-HRMS (*m/z*): [M + H]^+^ calcd for C_33_H_34_ClN_8_O_7_S 721.1954, obsd 721.1952.

##### 4-((1-(2-((5-Chloro-2-((4-(morpholinomethyl)phenyl)amino)pyrimidin-4-yl)amino)benzamido)propan-2-yl)oxy)-3-(phenylsulfonyl)-1,2,5-oxadiazole 2-oxide (**9c**)

As a yellow solid, 14% yield. mp 187–189 °C. ^1^H NMR (400 MHz, DMSO-*d*_6_) *δ* 11.29 (s, 1H), 9.44 (s, 1H), 8.93 (t, *J* = 5.2 Hz, 1H), 8.74 (d, *J* = 7.2 Hz, 1H), 8.20 (s, 1H), 7.96 (d, *J* = 8.1 Hz, 2H), 7.81–7.70 (m, 2H), 7.64–7.54 (m, 4H), 7.51 (t, *J* = 7.6 Hz, 1H), 7.24–7.15 (m, 3H), 5.22–5.11 (m, 1H), 3.76 (t, *J* = 4.0 Hz, 1H), 3.72 (t, *J* = 4.4 Hz, 1H), 3.58 (t, *J* = 4.0 Hz, 4H), 3.43 (s, 2H), 2.37 (br s, 4H), 1.42 (d, *J* = 4.0 Hz, 3H). ^13^C NMR (101 MHz, DMSO-*d*_6_) *δ* 169.48, 158.91, 158.21, 155.41, 155.07, 139.77, 139.60, 137.63, 136.38, 132.12, 130.22, 129.63, 128.81, 128.69, 122.41, 121.98, 121.19, 120.04, 111.11, 105.51, 78.17, 66.60, 62.49, 53.50, 43.28, 17.69. ESI-HRMS (*m/z*): [M + H]^+^ calcd for C_33_H_34_ClN_8_O_7_S 721.1954, obsd 721.1951.

##### 4-(2-(2-((5-Chloro-2-((4-(morpholinomethyl)phenyl)amino)pyrimidin-4-yl)amino)-*N*-methylbenzamido)ethoxy)-3-(phenylsulfonyl)-1,2,5-oxadiazole 2-oxide (**9d**)

As a yellow solid, 25% yield. mp 169–171 °C. ^1^H NMR (400 MHz, DMSO-*d*_6_) *δ* 9.34 (s, 1H), 8.99 (s, 1H), 8.15 (d, *J* = 8.0 Hz, 1H), 8.13 (s, 1H), 7.97 (d, *J* = 7.6 Hz, 2H), 7.85–7.78 (m, 1H), 7.75–7.63 (m, 2H), 7.54 (t, *J* = 7.6 Hz, 2H), 7.50 (d, *J* = 7.6 Hz, 2H), 7.32–7.20 (m, 1H), 7.11 (d, *J* = 8.1 Hz, 2H), 4.64 (br s, 2H), 3.93 (br s, 2H), 3.57 (t, *J* = 4.0 Hz, 4H), 3.40 (s, 2H), 3.05 (s, 3H), 2.35 (br s, 4H). ^13^C NMR (101 MHz, DMSO-*d*_6_) *δ* 169.80, 159.31, 158.17, 156.07, 154.98, 139.74, 137.58, 136.70, 136.56, 130.43, 129.57, 128.70, 128.39, 128.05, 125.95, 125.22, 124.16, 119.57, 111.07, 104.59, 69.64, 66.54, 62.43, 53.45, 46.28, 39.09. ESI-HRMS (*m/z*): [M + H]^+^ calcd for C_33_H_34_ClN_8_O_7_S 721.1954, obsd 721.1935.

##### 4-((1-(2-((5-Chloro-2-((4-(morpholinomethyl)phenyl)amino)pyrimidin-4-yl)amino)benzoyl)piperidin-4-yl)oxy)-3-(phenylsulfonyl)-1,2,5-oxadiazole 2-oxide (**9e**)

As a yellow solid, 19% yield. mp 130–132 °C. ^1^H NMR (400 MHz, DMSO-*d*_6_) *δ* 9.38 (s, 1H), 9.00 (s, 1H), 8.19 (s, 1H), 8.14 (d, *J* = 8.0 Hz, 1H), 8.01 (d, *J* = 7.6 Hz, 2H), 7.86 (t, *J* = 7.4 Hz, 1H), 7.72 (t, *J* = 7.8 Hz, 2H), 7.51 (t, *J* = 8.0 Hz, 3H), 7.46 (d, *J* = 7.3 Hz, 1H), 7.30 (t, *J* = 7.5 Hz, 1H), 7.09 (d, *J* = 8.3 Hz, 2H), 5.16–5.00 (m, 1H), 3.67 (br s, 2H), 3.62–3.51 (m, 5H), 3.37 (s, 3H), 2.32 (br s, 4H), 2.00 (br s, 1H), 1.84 (br s, 2H), 1.64 (br s, 1H). ^13^C NMR (101 MHz, DMSO-*d*_6_) *δ* 168.25, 158.22, 156.23, 155.10, 139.61, 137.71, 136.71, 136.54, 130.99, 130.47, 130.10, 129.45, 128.85, 128.34, 128.06, 125.60, 124.58, 119.58, 110.88, 104.47, 77.51, 66.82, 66.64, 62.53, 53.52, 29.51. ESI-HRMS (*m/z*): [M + H]^+^ calcd for C_35_H_36_ClN_8_O_7_S 747.2111, obsd 747.2092.

##### 4-((1-(2-((5-Chloro-2-((4-(morpholinomethyl)phenyl)amino)pyrimidin-4-yl)amino)benzoyl)piperidin-4-yl)methoxy)-3-(phenylsulfonyl)-1,2,5-oxadiazole 2-oxide (**9f**)

As a yellow solid, 29% yield. mp 109–111 °C. ^1^H NMR (400 MHz, DMSO-*d*_6_) *δ* 9.36 (s, 1H), 8.99 (s, 1H), 8.16 (d, *J* = 7.8 Hz, 1H), 8.14 (s, 1H), 7.98 (d, *J* = 7.2 Hz, 2H), 7.84 (t, *J* = 7.5 Hz, 1H), 7.70 (t, *J* = 7.8 Hz, 2H), 7.53 (d, *J* = 8.4 Hz, 1H), 7.50 (t, *J* = 8.4 Hz, 2H), 7.40 (d, *J* = 7.6 Hz, 1H), 7.28 (t, *J* = 7.5 Hz, 1H), 7.10 (d, *J* = 8.4 Hz, 2H), 4.53 (br s, 1H), 4.21 (d, *J* = 5.9 Hz, 2H), 3.70 (br s, 1H), 3.58 (t, *J* = 4.0 Hz, 4H), 3.38 (s, 2H), 3.03 (br s, 1H), 2.81 (br s, 1H), 2.33 (br s, 4H), 2.13–1.90 (m, 2H), 1.75 (br s, 1H), 1.60–1.42 (m, 2H). ^13^C NMR (101 MHz, DMSO-*d*_6_) *δ* 168.05, 159.25, 158.19, 156.05, 155.01, 139.65, 137.63, 136.74, 136.55, 130.47, 130.32, 129.51, 128.75, 128.20, 127.92, 125.24, 124.33, 119.58, 110.85, 104.56, 75.08, 70.25, 66.59, 62.48, 53.49, 52.77, 35.08, 29.55, 29.21. ESI-HRMS (*m/z*): [M + H]^+^ calcd for C_36_H_38_ClN_8_O_7_S 761.2267, obsd 761.2235.

##### 4-((6-(2-((5-Chloro-2-((4-(morpholinomethyl)phenyl)amino)pyrimidin-4-yl)amino)benzamido)hexyl)oxy)-3-(phenylsulfonyl)-1,2,5-oxadiazole 2-oxide (**9g**)

As a yellow solid, 30% yield. mp 120–122 °C. ^1^H NMR (400 MHz, DMSO-*d*_6_) *δ* 11.46 (s, 1H), 9.48 (s, 1H), 8.80 (t, *J* = 5.4 Hz, 1H), 8.72 (d, *J* = 7.8 Hz, 1H), 8.20 (s, 1H), 8.02 (d, *J* = 7.5 Hz, 2H), 7.88 (t, *J* = 7.5 Hz, 1H), 7.79–7.73 (m, 3H), 7.62 (d, *J* = 8.3 Hz, 2H), 7.48 (t, *J* = 7.6 Hz, 1H), 7.21 (d, *J* = 8.4 Hz, 2H), 7.17 (t, *J* = 7.2 Hz, 1H), 4.38 (t, *J* = 6.2 Hz, 2H), 3.59 (t, *J* = 4.4 Hz, 4H), 3.45 (s, 2H), 3.32 (t, *J* = 6.2 Hz, 2H), 2.39 (br s, 4H), 1.75 (quint, *J* = 5.6 Hz, 2H), 1.58 (quint, *J* = 6.1 Hz, 2H), 1.42–1.34 (m, 4H). ^13^C NMR (101 MHz, DMSO-*d*_6_) *δ* 168.89, 159.31, 158.22, 155.45, 155.04, 139.63, 139.58, 137.72, 136.56, 131.76, 130.47, 129.63, 128.74, 128.55, 122.46, 121.99, 121.89, 120.00, 110.87, 105.44, 71.85, 66.58, 62.49, 53.49, 29.24, 28.28, 26.42, 25.25. ESI-HRMS (*m/z*): [M + H]^+^ calcd for C_36_H_40_ClN_8_O_7_S 763.2424, obsd 763.2405.

### 3.2. In Vitro Kinase Enzymatic Assay

The ADP-Glo™ Kinase Assay Kit and FAK Kinase Enzyme (Catalog. V9301) were purchased from Promega Corporation (Madison, WI, USA). The experiments were performed according to the detailed and complete protocols of the manufacturer. Briefly, the concentration of FAK determined by optimization experiments was 0.1 μg/μL. Suitable concentration gradients from 1.6 to 1000 nM were set for all the tested compounds. A kinase reaction buffer, a substrate/ATP mixture, and a FAK solution were prepared right before use. The 5 μL kinase reaction consisted of 1 μL of compound solution, 2 μL of substrate/ATP mixture, and 2 μL of FAK solution. The control without enzyme was provided by 3 μL of kinase reaction buffer and 2 μL of substrate/ATP mixture. The negative control was prepared using 1 μL of kinase reaction buffer instead of a compound solution. The kinase reactions were added to a 384-well plate and incubated at room temperature for 60 min, then 5 μL of ADP-Glo™ Reagent was added to stop the kinase reaction and deplete the unconsumed ATP, and incubated at room temperature for 40 min. After adding 10 μL of Kinase Detection Reagent, the plate was incubated at room temperature for 30 min and then luminescence was recorded (integration time 0.5–1 s) on a TriStar^®^ LB942 Multimode Microplate Reader (BERTHOLD TECHNOLOGIES GmbH & Co. KG., Bad Wildbad, Germany). Curve fitting and data presentations were performed using GraphPad Prism version 8.0.

### 3.3. Cell Viability Determination

The H1975 human NSCLC cell line was obtained from ATCC. A431 human epithelial carcinoma and HBE human bronchial epithelial cell lines were kind gifts from Fuheng Biological Company Ltd. (Shanghai, China) and YuXi Biotech Company (Jiangsu, China), respectively. The cells were maintained in RPMI 1640 or DMEM (Gibco^®^, Thermo Fisher Scientific Inc., Waltham, MA, USA) containing 10% fetal bovine serum (FBS, Gibco^®^) and 1% penicillin-streptomycin (Beyotime Company, Shanghai, China) at 37 °C in a 5% CO_2_ incubator.

A total of 3000 cells of H1975 or HBE and 5000 cells of A431 were seeded for each well into a 96-well plate and cultured in 10% FBS respective growth medium overnight. Various concentrations (0–20 μM) of each compound were then added in triplicate to the cells. After 72 h of incubation, 10 μL of CCK-8 solution (5.0 mg/mL, Biotool Company, Kirchberg, Switzerland) was added to each well and the cells continued to culture for 4 h. The optical density (OD) was determined at 450 nm with a microplate reader (Thermo Fisher Scientific, Waltham, MA, USA). IC_50_ values were calculated employing GraphPad Prism version 8.0.

### 3.4. Acridine Orange/Ethidium Bromide (AO/EB) Staining Assay

H1975 cells at a density of 1 × 10^5^ cells/well in 6-well plates were incubated for 24 h. After discarding the original culture medium, different concentrations of inhibitors were used. The cells were incubated for 48 h and then washed with phosphate-buffered saline (PBS) three times. Cells were subsequently incubated in the dye mix containing 1.0 µg/mL of AO and 1.0 µg/mL of EB in PBS at 37 °C for 5 min, and then washed with PBS. The apoptotic, necrotic, and live cells were examined under the fluorescent inverted microscope (OLYMPUS, Tokyo, Japan).

### 3.5. Flow Cytometric Analysis

H1975 cells were seeded at a density of 1 × 10^5^ cells/well into 6-well plates for 24 h and then exposed to different concentrations of inhibitors for 48 h. The cells were harvested. (1) The cells were stained with Annexin V-FITC (5 µL)/propidium iodide (5 µL), and the samples were detected and flow cytometry (Becton-Dickinson, Franklin Lakes, NJ, USA) was performed to quantify the number of apoptotic cells. (2) The cells were stained with propidium iodide (PI) in the dark for 30 min, and then analyzed for cell cycle distribution by the flow cytometry assay.

### 3.6. Scratch Assay

H1975 cells were seeded in 6-well plates and incubated for 12 h. When cells were grown to 90–100% confluency in a monolayer, wounds were generated using a pipette tip. The cells were washed three times with PBS, treated with different concentrations of compound **8a** or TAE226, and incubated for 24 h. The images were captured to record the status of cells near the scratch lines at the beginning and after 12 and 24 h.

### 3.7. Molecular Docking Study

Molecular docking simulations were carried out on the Discovery Studio 2017 R2 software (Dassault Systèmes BIOVIA, San Diego, CA, USA). The crystal structure of the FAK (PDB: 2JKK) in complex with TAE226 was used and the corresponding PDB files were directly loaded into the Discovery Studio 2017 R2 software. The FAK kinase was prepared using the “Prepare Protein” plugin, while the structure of ligand **8a** was optimized using the “Prepare Ligands” plugin. The binding site in FAK was defined from the current selection when TAE226 was selected. Docking was performed using CDOCKER, while the parameter for “Pose Cluster Radius” was set as 0.5 and the remaining parameters were at default settings. The best predicted hit with the highest docking score was used for the visualization with 2JKK.

### 3.8. Statistical Analysis

All statistical analyses were performed using the GraphPad Prism software version 8.0 (GraphPad Software Inc., La Jolla, CA, USA). Statistically significant differences were analyzed using the unpaired t tests, and *p*-values of less than 0.05 were considered to indicate statistical significance.

## 4. Conclusions

In summary, a series of 2,4-dianilinopyrimidine derivatives **8a**–**8i** and **9a**–**9g** bearing 4-(morpholinomethyl)phenyl and *N*-substituted benzamides were designed and synthesized. Many of these derivatives inhibited FAK at low-nanomolar concentrations. In particular, compounds **8a**, **8c**, and **8d** exhibited the highest inhibition against FAK compared to other compounds. The antiproliferative effects of compounds **8a**, **8b**, and **9a** against H1975 and A431 cells were stronger than both TAE226 and osimertinib; however, most of the target compounds weakly inhibited the growth of HBE cells compared to H1975 cells. Compound **8a** demonstrated the best combination of potency and selectivity against tumor cells compared to the other compounds. Unexpectedly, compounds **8a**–**8g** featuring hydroxyl groups displayed higher potencies against FAK and antiproliferative activities compared to compounds **9a**–**9g** bearing furoxan moieties. Therefore, the introduction of furoxan did not improve the inhibitory activity of the scaffold against FAK or the growth of the cancer cells. In the further studies of compound **8a** using H1975 cells, **8a** induced cell apoptosis in a dose-dependent manner, causing S/G2 arrest and inhibiting cell migration, all of which were superior to TAE226. Molecular docking studies demonstrated that **8a** was bound in the active pocket of FAK in a conformationally similar manner compared to TAE226, but two additional hydrogen bonds were formed between **8a** and the active site residues. Overall, these results suggested that compound **8a** might be a potential inhibitor of FAK for treatment of various cancers, although further investigation is required to evaluate the efficacy in vivo.

## Figures and Tables

**Table 1:** Biological activities of compounds **8a**–**8i** and **9a**–**9g**.

Compound	R	FAK, IC_50_ (μM) *^a^*	Antiproliferative Activity IC_50_ (μM) *^a^*			SI Values *^b^*
			H1975	A431	HBE	
**8a**	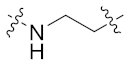	0.047 ± 0.006	0.044 ± 0.011	0.119 ± 0.036	1.039 ± 0.266	24
**8b**	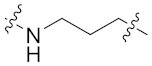	0.061 ± 0.009	0.075 ± 0.011	0.207 ± 0.036	1.153 ± 0.283	15
**8c**	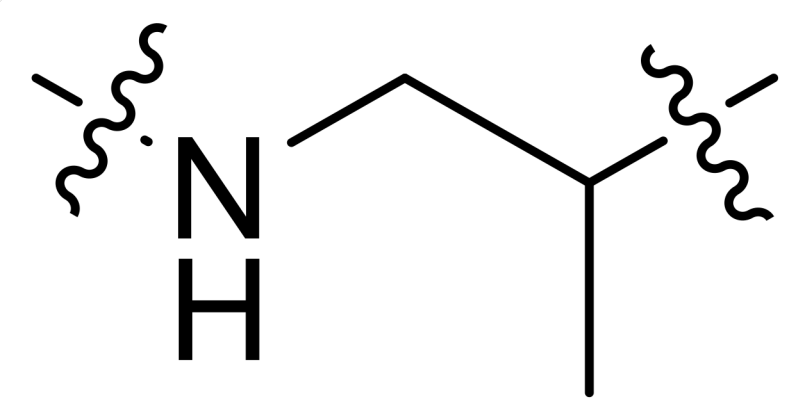	0.030 ± 0.007	0.119 ± 0.047	0.158 ± 0.027	4.007 ± 0.231	34
**8d**	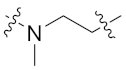	0.040 ± 0.011	0.293 ± 0.050	0.161 ± 0.082	3.825 ± 0.500	13
**8e**	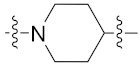	0.129 ± 0.037	1.414 ± 0.132	0.238 ± 0.098	0.435 ± 0.045	0.3
**8f**	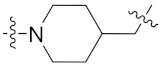	0.173 ± 0.023	2.221 ± 0.261	0.195 ± 0.097	0.862 ± 0.125	0.4
**8g**	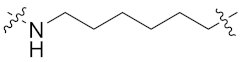	0.198 ± 0.032	0.287 ± 0.061	0.507 ± 0.019	1.645 ± 0.813	6
**8h**	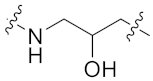	0.072 ± 0.007	0.219 ± 0.082	0.047 ± 0.006	0.688 ± 0.121	3
**8i**	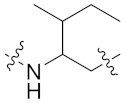	0.126 ± 0.021	0.815 ± 0.061	1.245 ± 0.909	>20	>24
**9a**	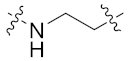	0.149 ± 0.033	0.047 ± 0.009	0.182 ± 0.032	0.916 ± 0.092	19
**9b**	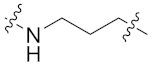	0.338 ± 0.038	0.164 ± 0.051	0.138 ± 0.031	2.078 ± 0.366	13
**9c**	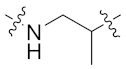	0.089 ± 0.014	0.567 ± 0.086	1.074 ± 0.154	2.418 ± 0.546	4
**9d**	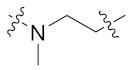	0.066 ± 0.014	1.094 ± 0.137	2.738 ± 0.206	4.552 ± 0.533	4
**9e**	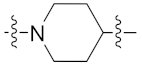	>1	1.724 ± 0.295	2.414 ± 0.587	0.763 ± 0.100	0.4
**9f**	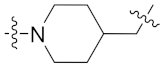	0.300 ± 0.009	1.570 ± 0.265	1.739 ± 0.305	3.761 ± 1.604	2
**9g**	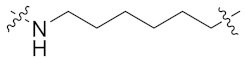	>1	0.592 ± 0.073	0.617 ± 0.040	3.009 ± 0.090	5
**TAE226**		0.0034 ± 0.0005	0.141 ± 0.041	0.392 ± 0.053	>20	>142
**Osimertinib**		ND	0.116 ± 0.032	0.988 ± 0.119	1.208 ± 0.177	10

*^a^* IC_50_ data were means ± SD of three independent experiments. *^b^* Selectivity index (SI) value = IC_50_ for HBE cell line/IC_50_ for H1975 cell line. ND, not determined.

## Data Availability

Data is contained within the article.

## Sample Availability

Samples of the compounds are available from the authors.
