# Correction: Han et al. Discovery of Novel 2,4-Dianilinopyrimidine Derivatives Containing 4-(Morpholinomethyl)phenyl and *N*-Substituted Benzamides as Potential FAK Inhibitors and Anticancer Agents. *Molecules* 2021, *26*, 4187

**DOI:** 10.3390/molecules31193366

**Published:** 2026-09-22

**Authors:** Chun Han, Kemin Shen, Shijun Wang, Zhijun Wang, Feng Su, Xi Wu, Xiaoqin Hu, Mengyao Li, Jing Han, Lintao Wu

**Affiliations:** 1 Department of Chemistry, Changzhi University, Changzhi 046011, China; coldspringfibre@126.com (C.H.); 444795415@nwafu.edu.cn (S.W.); czxywzj@czc.edu.cn (Z.W.); sufeng@czc.edu.cn (F.S.); wuxi040252768@163.com (X.W.); xiaoqin_hu2021@126.com (X.H.); lmysummer@126.com (M.L.); 2 Department of Public Health and Preventive Medicine, Changzhi Medical College, Changzhi 046011, China; shenkm@czmc.edu.cn; 3 School of Chemistry and Materials Science, Jiangsu Normal University, Xuzhou 221116, China

**Error in Figure**

In the original publication [1], there was a mistake in Figure 9 as published. During the preparation of the manuscript, microscopy imaging and layout issues led to several images in Figure 9 being inadvertently presented in a form that did not accurately represent the original images. The corrected Figure 9, obtained from a parallel experiment conducted under the same conditions and using the same batch of samples, appears below. The authors state that the scientific conclusions are unaffected. This correction was approved by the Academic Editor. The original publication has also been updated.

## Figures and Tables

**Figure 9:**
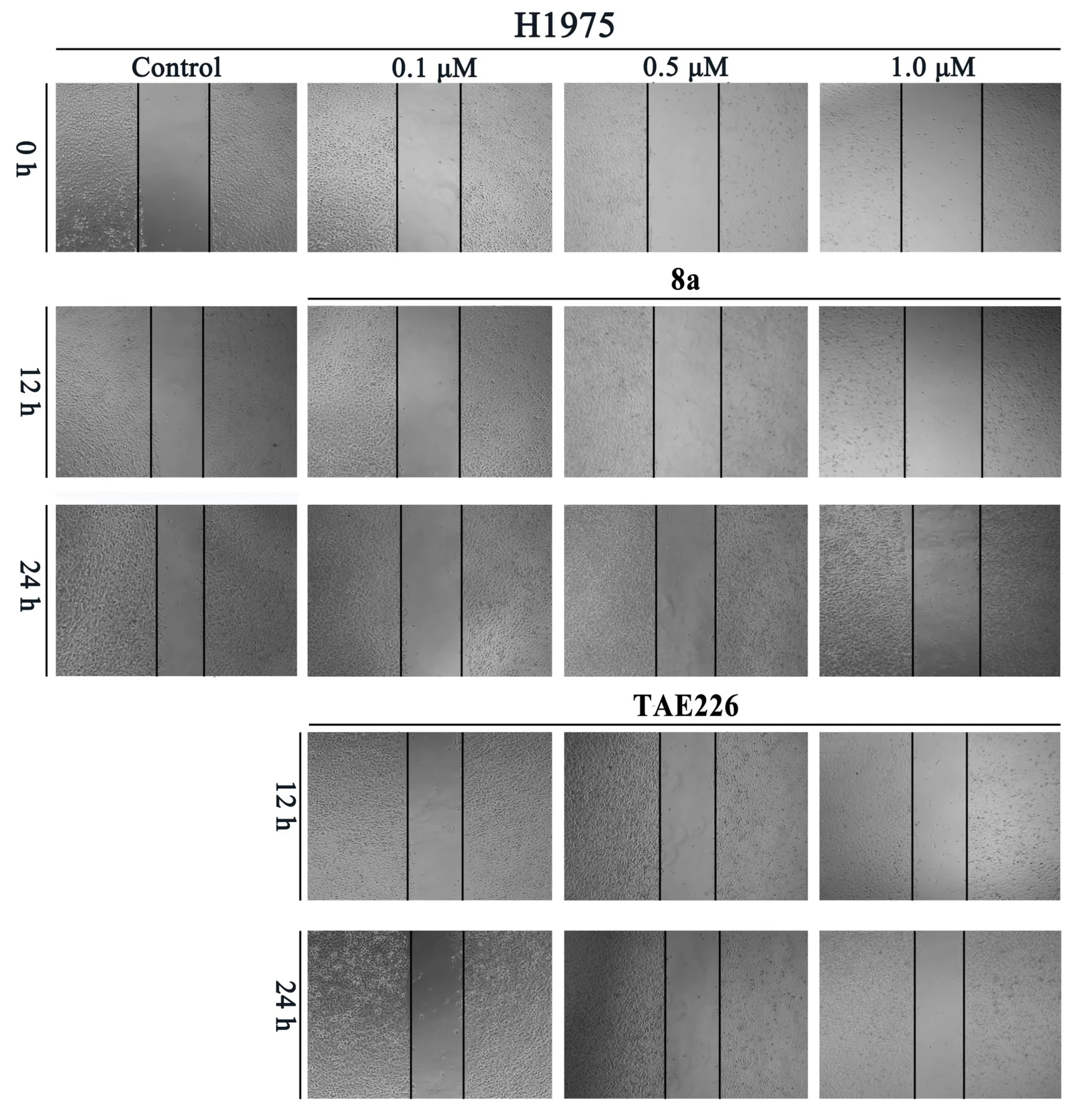
Effects of compound **8a** and TAE226 on the migration of H1975 cells in vitro determined by the scratch assay. H1975 cells were incubated with different concentrations of compound **8a** and TAE226 (0.1, 0.5, and 1 μM) for 24 h.
